# Structural mechanism of cooperative activation of the human calcium-sensing receptor by Ca^2+^ ions and L-tryptophan

**DOI:** 10.1038/s41422-021-00474-0

**Published:** 2021-02-18

**Authors:** Shenglong Ling, Pan Shi, Sanling Liu, Xianyu Meng, Yingxin Zhou, Wenjing Sun, Shenghai Chang, Xing Zhang, Longhua Zhang, Chaowei Shi, Demeng Sun, Lei Liu, Changlin Tian

**Affiliations:** 1 Hefei National Laboratory of Physical Sciences at Microscale, Anhui Laboratory of Advanced Photonic Science and Technology, and School of Life Sciences, University of Science and Technology of China, Hefei, Anhui 230026 China; 2 School of Medicine, Zhejiang University, Hangzhou, Zhejiang 310011 China; 3 Tsinghua-Peking Joint Center for Life Sciences, Ministry of Education Key Laboratory of Bioorganic Phosphorus Chemistry and Chemical Biology, Department of Chemistry, Tsinghua University, Beijing, 100084 China; 4 High Magnetic Field Laboratory, Chinese Academy of Sciences, Hefei, Anhui 230030 China; 5 Shanghai Institute of Materia Medica, Chinese Academy of Sciences, Shanghai, 201203 China

**Keywords:** Cryoelectron microscopy, Calcium signalling

## Abstract

The human calcium-sensing receptor (CaSR) is a class C G protein-coupled receptor (GPCR) responsible for maintaining Ca^2+^ homeostasis in the blood. The general consensus is that extracellular Ca^2+^ is the principal agonist of CaSR. Aliphatic and aromatic L-amino acids, such as L-Phe and L-Trp, increase the sensitivity of CaSR towards Ca^2+^ and are considered allosteric activators. Crystal structures of the extracellular domain (ECD) of CaSR dimer have demonstrated Ca^2+^ and L-Trp binding sites and conformational changes of the ECD upon Ca^2+^/L-Trp binding. However, it remains to be understood at the structural level how Ca^2+^/L-Trp binding to the ECD leads to conformational changes in transmembrane domains (TMDs) and consequent CaSR activation. Here, we determined the structures of full-length human CaSR in the inactive state, Ca^2+^- or L-Trp-bound states, and Ca^2+^/L-Trp-bound active state using single-particle cryo-electron microscopy. Structural studies demonstrate that L-Trp binding induces the closure of the Venus flytrap (VFT) domain of CaSR, bringing the receptor into an intermediate active state. Ca^2+^ binding relays the conformational changes from the VFT domains to the TMDs, consequently inducing close contact between the two TMDs of dimeric CaSR, activating the receptor. Importantly, our structural and functional studies reveal that Ca^2+^ ions and L-Trp activate CaSR cooperatively. Amino acids are not able to activate CaSR alone, but can promote the receptor activation in the presence of Ca^2+^. Our data provide complementary insights into the activation of class C GPCRs and may aid in the development of novel drugs targeting CaSR.

## Introduction

Calcium-sensing receptor (CaSR) is a class C G protein-coupled receptor (GPCR) that regulates Ca^2+^ homeostasis by monitoring extracellular levels of Ca^2+^ ions.^1–3^ CaSR responds to a diverse array of stimuli, such as cations, amino acids, polyamines, and polypeptides.^2–6^ Malfunctions in CaSR have been correlated with hypercalcaemic and hypocalcaemic disorders, such as familial hypocalciuric hypercalcaemia (FHH), neonatal severe hyperparathyroidism (NSHPT), and autosomal dominant hypocalcaemic hypercalciuria (ADH).^7,8^ CaSR is a homodimer,^9,10^ and each subunit possesses a bi-lobed Venus flytrap (VFT) domain-containing orthosteric binding sites for native ligands and a cysteine-rich domain (CRD) linking the VFT domain to the seven-helix transmembrane domain (TMD).^4,11–13^ Mutagenesis, biochemistry, and functional studies have been conducted to illustrate the molecular basis of intracellular inositol phosphate 1 (IP_1_) accumulation upon agonist activation of CaSR and the mechanisms of human diseases arising from CaSR mutation.^7,14,15^ Recently, using a cell-free Förster resonance energy transfer (FRET)-based conformational CaSR biosensor, it was reported that Ca^2+^ alone fully stabilizes the active conformation, while amino acids behave as pure positive allosteric modulators.^14^ Crystal structures of the CaSR extracellular domain (ECD) have shown that Ca^2+^ and L-tryptophan (L-Trp) act as co-agonists to induce receptor activation.^4^ It was also reported that Mg^2+^ and an unexpected tryptophan derivative (L-1,2,3,4-tetrahydronorharman-3-carboxylic acid, TNCA) are able to activate CaSR cooperatively.^11^ Pronounced conformational changes occurred in the VFT domain of the CaSR ECD upon agonist binding, leading to a change in the orientation between its two lobes (LB1 and LB2) and a consequent conformational switch from an open to a closed conformation.^4^ This agonist binding-induced conformational change in the VFT domain was also observed in other class C GPCRs, such as mGluR1, mGluR5, and GABA_B_.^16–23^ Furthermore, structural studies of full-length mGluR5 and GABA_B_ have illustrated that agonist-induced conformational changes in the VFT domains can lead to rearrangements of the helices within the TMD, facilitating G protein binding and intracellular signal transduction.^17,20,21^ However, the structure of full-length CaSR has not yet been reported. Structural insights into the activation of full-length CaSR involving the TMD remain elusive. A convincing mechanism of signal transduction from the ECD to the TMD of CaSR remains to be determined.

In this study, we determined the structures of full-length human CaSR in the inactive state, L-Trp- or Ca^2+^-bound and L-Trp/Ca^2+^-bound states using single-particle cryo-electron microscopy (cryo-EM). Cryo-EM structures of CaSR in an inactive state revealed conformational heterogeneity of the receptor. The dimeric VFT domains were observed to adopt open-open, open-closed, or closed-closed conformations. Interestingly, our structural and functional data indicate that L-Trp binding results in the closure of open VFT domains, inducing them to adopt a homogeneous closed conformation and promoting a conformational switch of CaSR from an open-open to a closed-closed conformation. The L-Trp-bound closed-closed conformation is therefore considered to be an intermediate state of CaSR during its activation. Ca^2+^ is able to push CaSR proteins in an inactive closed-closed (Icc) state into an active closed-closed (Acc) state through domain twisting. The CRDs and TMDs in active CaSR are in close proximity, unlike the separated architectures observed in the structure of inactive CaSR. The consequent rotation and rearrangement of TMDs promote the transmembrane bundle, forming a new interface mediated by TM6, which provides a structural basis for G protein recruitment and signal transduction. Thus, this study clearly illustrates the roles of Ca^2+^ and L-Trp in the activation of CaSR, provides new structural insights into the activation of class C GPCRs and extends our knowledge of diverse G protein-mediated signal transduction by class C GPCRs upon agonist activation.

## Results

### Cryo-EM structure of CaSR in the active state

**Fig. 1:**
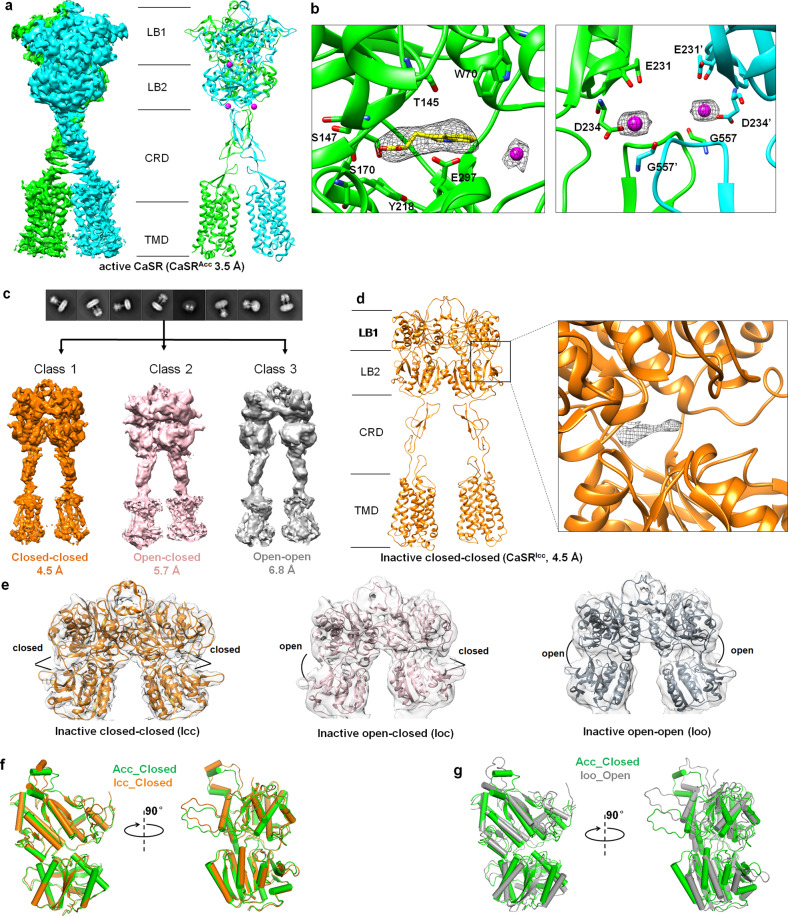
Cryo-EM structures of CaSR in active and inactive states. **a** Map and cartoon representations of the dimeric CaSR structure in the Ca^2+^/L-Trp-bound state, representing the structure of active CaSR. The two subunits are colored in green and cyan, respectively. Ca^2+^ ions are shown as magenta spheres. **b** Detailed view of the L-Trp and Ca^2+^ binding sites of CaSR in the active CaSR structure. L-Trp is shown as yellow stick, and Ca^2+^ is shown as magenta sphere. Residues in CaSR that participate in the interaction with Ca^2+^ and L-Trp are shown as sticks. **c** Representative 2D class average images (upper panel) and three distinct classes of 3D reconstruction density maps are obtained with resolutions of 4.5 Å, 5.7 Å, and 6.8 Å, respectively (lower panel). **d** Cartoon representation of the dimeric inactive CaSR structure in the Icc conformation (CaSR^Icc^). Extra density located at the cleft between LB1 and LB2 in the VFT domain is shown in the inset. **e** Corresponding coordinates fit into the reconstruction maps of the LB1/LB2 of each VFT domain in the three models shown in **c**. The dimeric VFT domains with Icc conformation are shown in orange, and Ioc and Ioo VFT domains are shown in wheat and gray, respectively. **f** Superimposition of the closed VFT domains of CaSR in the Acc and Icc conformations, showing the structural similarity of the two closed VFT domains. **g** Superimposition of a single VFT domain in Acc and Ioo conformations, showing the opening of the VFT in the Ioo conformation (gray) and the closure of the VFT in the Acc conformation (green).

The full-length human CaSR protein was overexpressed in Sf9 cells and purified in the presence of DDM/CHS (*n*-dodecyl-β-D-maltoside/3β-hydroxy-5-cholestene-3-hemisuccinate) (Supplementary information, Fig. S1a). To obtain the CaSR protein in an active state, 10 mM Ca^2+^ and 10 mM L-Trp were supplemented throughout the protein purification and sample preparation stages. The sample was subjected to single-particle cryo-EM (Supplementary information, Fig. S1b, c). Finally, we obtained a 3D density map of Ca^2+^/L-Trp-bound CaSR with an overall resolution of 3.5 Å (Supplementary information, Fig. S1d–g). The density map could demonstrate secondary structure features, particularly for the majority of α-helices within the VFT domain and TMD layers of the CaSR, enabling us to readily build the structural model for CaSR in the Ca^2+^/L-Trp-bound state (Fig. 1a; Supplementary information, Fig. S1 and Table S1). The VFT domain, CRD, and TMD were explicitly identified in the structure.

The cryo-EM structure of CaSR shows a dimeric architecture in which the two subunits are bound to each other in a side-by-side configuration (Fig. 1a). In each subunit, two Ca^2+^ ions and one L-Trp molecule were assigned according to the extra density in the map. The first Ca^2+^ ion is located in the cleft between LB1 and LB2 of the VFT domain (Fig. 1b). The second Ca^2+^ ion resides at the LB2–CRD interface, bridging the interaction between the LB2 region and CRD of the adjacent subunit. The L-Trp molecule occupies the cleft of the VFT domain, located close to the first Ca^2+^ ion. Residues W70, T145, S147, and S170 in LB1, Y218, and E297 in LB2 are observed to contact L-Trp (Fig. 1b), consistent with those observed in the crystal structure of CaSR-ECD in complex with L-Trp.^4^ In each subunit of CaSR, six *N*-linked glycans are observed to conjugate to the receptor at glycosylation sites N261, N287, N446, N468, N488 and N541 (Supplementary information, Fig. S2), consistent with the glycosylation sites reported previously.^4^ These post-translational modifications could play important roles in receptor trafficking, cellular surface expression, and functions.^4,24^

Overall, the active CaSR dimer adopts a compact architecture, which is similar to that of mGluR5 in an agonist-bound active state^17^ (Supplementary information, Fig. S3a). In particular, the structure of the agonist-bound VFT domain derived from full-length active CaSR shows high similarity to those from active mGluR5 and truncated CaSR-ECD (RMSD = 1.40 Å and 0.63 Å, respectively) (Supplementary information, Fig. S3b, c). That is, Ca^2+^/L-Trp-bound CaSR adopts an Acc conformation (designated as CaSR^Acc^). The compact architecture of CaSR^Acc^ is maintained by multiple intersubunit interfaces between the VFT, CRD, and TMD of each subunit (Supplementary information, Fig. S4). In addition to the previously reported interfaces in the LB1 and LB2 regions of the VFT domain and in the CRD,^4^ our structure clearly reveals that the two transmembrane bundles of the two subunits contact each other through the C-terminus of TM6 in the active CaSR (Supplementary information, Fig. S4h). Interactions between the second extracellular loops (ECL2) of the two TMDs are also observed (Supplementary information, Fig. S4g).

### Cryo-EM structures of CaSR in the inactive state reveal conformational heterogeneity

We also determined the structure of CaSR in an inactive state. EDTA was utilized to remove trace Ca^2+^ and other divalent cations (such as Mg^2+^) throughout the protein purification process (Supplementary information, Fig. S5a). The Ca^2+^-free sample, representing CaSR in an inactive state, was then subjected to cryo-EM (Supplementary information, Fig. S5b, c). After extensive 3D classification, three models with well-sampled Euler angle distributions were obtained. The final 3D refinement maps of the three models have overall resolutions of 4.5 Å, 5.7 Å, and 6.8 Å, respectively (Supplementary information, Fig. S5d–g). These models show similar architectures: an inverted V-shaped dimer in which the two subunits are bound to each other through the VFT domain in a side-by-side configuration (Fig. 1c). Despite this, the density maps of the models revealed striking differences in the dimeric VFT domains. The VFT domains of the three models were shown to adopt closed-closed, open-closed, and open-open conformations (Fig. 1e; Supplementary information, Fig. S6a), indicating substantial conformational heterogeneity of CaSR in an inactive state. We designated the three models as CaSR^Icc^ (4.5 Å), inactive open-closed (CaSR^Ioc^, 5.7 Å), and inactive open-open (CaSR^Ioo^, 6.8 Å) conformations of CaSR. The density map of CaSR^Icc^ has a relatively higher resolution, allowing us to build the structure of full-length CaSR in the Icc conformation (Fig. 1d; Supplementary information, Fig. S7).

The striking conformational heterogeneity of CaSR in an inactive state is different from the relatively homogeneous distribution of other class C GPCRs in the apo-form inactive state.^17,20–23^ The open conformation of VFT is thought to be a common feature of class C GPCRs in an inactive state and has been observed in the structures of truncated CaSR-ECD and full-length mGluR5 and GABA_B_ in an inactive state.^17–23^ Comparing the structure of the open VFT domain of CaSR^Ioo^ with those of CaSR-ECD, mGluR5, and GABA_B_ revealed that the open conformations of the VFT domains are similar, while the angles between LB1 and LB2 in the VFT domains of full-length CaSR, CaSR-ECD, and mGluR5 are not exactly the same (Supplementary information, Fig. S6b–d). However, the closed conformations of VFT domains observed in the structures of CaSR^Icc^ and CaSR^Ioc^ are unexpected and rarely reported. Interestingly, structural alignment of the VFT domain derived from CaSR^Icc^ and CaSR^Acc^ gave an RMSD value of 0.74 Å, indicating a high similarity between the structures of the two VFT domains (Fig. 1f). In contrast, the structures of VFT domains from CaSR^Ioo^ and CaSR^Acc^ are significantly different (Fig. 1g).

Having a closer look at the density map of CaSR^Icc^, we found clear extra densities in both VFT domains. The density was observed to be located in the cleft between LB1 and LB2 and was far above the noise level, indicating that the corresponding positions are occupied by some small molecules (Fig. 1d). It has been reported that amino acids, such as L-Phe and L-Trp, and tryptophan derivative ligands can bind to CaSR and contribute to activation of the receptor.^4,6,11,25^ We suspect that the ambient amino acid or its derivative might already bind to CaSR during the expression stage and cannot be eliminated during purification. The binding of ambient amino acids at the VFT domain could result in the closed conformation of the VFT domain in CaSR^Icc^.

### Structure of the CaSR–L-Trp complex reveals an intermediate state of CaSR during activation

To verify that the binding of ambient amino acids or their derivatives at the VFT domain results in the closed conformation of the VFT domain in inactive CaSR, we set out to determine the structure of CaSR in the presence of L-Trp and in the absence of Ca^2+^. The purified, Ca^2+^-free CaSR (EGTA-treated) protein samples were supplemented with excess L-Trp (10 mM) and subjected to cryo-EM. After extensive 3D classification, only one model with an overall resolution of 4.4 Å was obtained, showing a conformational homogeneity of CaSR in the presence of L-Trp (Supplementary information, Fig. S8).

**Fig. 2:**
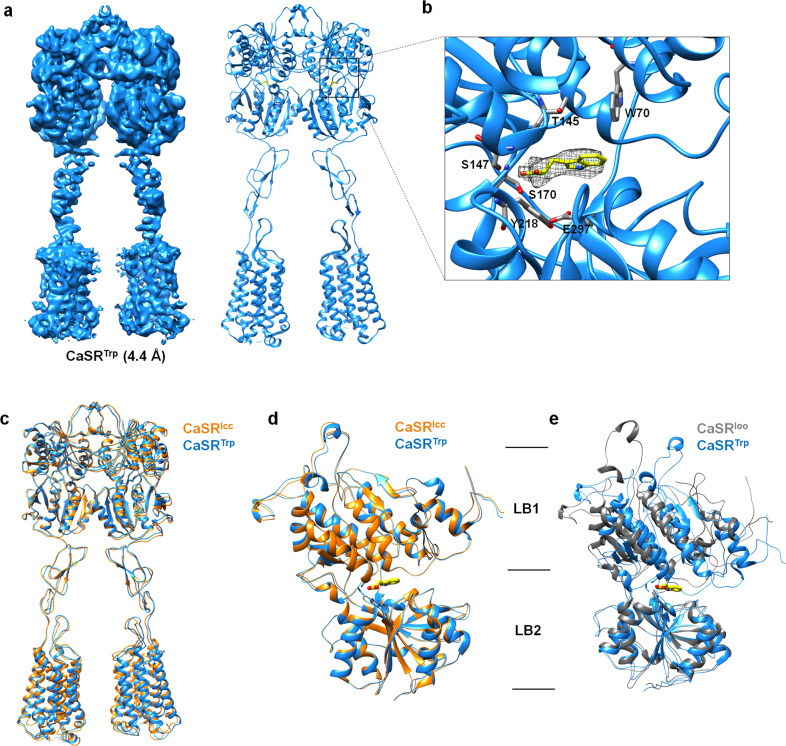
Cryo-EM structure of CaSR in the L-Trp-bound state. **a** Map and cartoon representations of the dimeric CaSR structure in the L-Trp-bound state (CaSR^Trp^). The two subunits of CaSR are colored in cyan. L-Trp molecules are shown as yellow sticks. **b** Cartoon representation of a single VFT domain structure of CaSR^Trp^. The density that was assigned to L-Trp is shown as mesh. **c** Superposition of the overall structures of CaSR^Trp^ and CaSR in the Icc conformation, indicating high structural similarity. **d** Structural superposition of single VFT domains derived from CaSR^Trp^ (blue) and CaSR^Icc^ (orange). The VFT domains from CaSR in both CaSR^Trp^ and CaSR^Icc^ reveal “closed” conformations. **e** Structural superimposition of single VFT domains in CaSR^Trp^ and CaSR^Ioo^, showing the opening of the VFT in the Ioo conformation (gray) and the closure of the VFT in the CaSR^Trp^ (blue).

The 4.4 Å map allowed us to build a structural model of CaSR in complex with L-Trp (designated as CaSR^Trp^) (Fig. 2a; Supplementary information, Fig. S9). In each subunit of dimeric CaSR^Trp^, one L-Trp molecule was clearly assigned according to the density in the map, which is located at the cleft between LB1 and LB2 of the VFT domain (Fig. 2b). We found that the structures of dimeric CaSR^Trp^ and CaSR^Icc^ are superimposed very well (Fig. 2c). In particular, structural alignment of a single subunit of CaSR^Trp^ with that of CaSR^Icc^ gave an RMSD value of 0.62 Å, and structural alignment of the single VFT domain derived from CaSR^Trp^ and CaSR^Icc^ gave an RMSD value of 0.41 Å (Fig. 2d). These observations indicate that the structures of CaSR^Trp^ and CaSR^Icc^ are identical. CaSR^Icc^ actually represents the structure of CaSR in a ligand (amino acids or its derivative)-bound state rather than the “ligand-free” state. However, due to the low resolution, the ligand in CaSR^Icc^ could not be identified.

Furthermore, it could be learned from the structures of mGluR5 and GABA_B_ that the open conformation of the VFT domain represents a typical structural feature of class C GPCRs in an apo-form inactive state.^17,20–23^ For CaSR, the structure of VFT in CaSR^Ioo^ adopts an open conformation different from that of CaSR^Trp^, but similar to those of mGluR5 and GABA_B_ in apo-form inactive states (Fig. 2e; Supplementary information, Fig. S6b, d). Therefore, we suggest that CaSR^Ioo^ represents the real “ligand-free” inactive state of CaSR, corresponding to the observed “resting open-open” (Roo) state of mGluR5.^17^ Structural investigations of CaSR^Ioo^ and CaSR^Icc^ offer an interesting inference: the L-Trp-bound Icc conformation could be an intermediate state of CaSR during activation. This intermediate state suggests that CaSR could be activated by its agonists through a novel mechanism, which has not been illustrated before.

### Cryo-EM structures of CaSR in the presence of a high concentration of Ca^2+^ ions

**Fig. 3:**
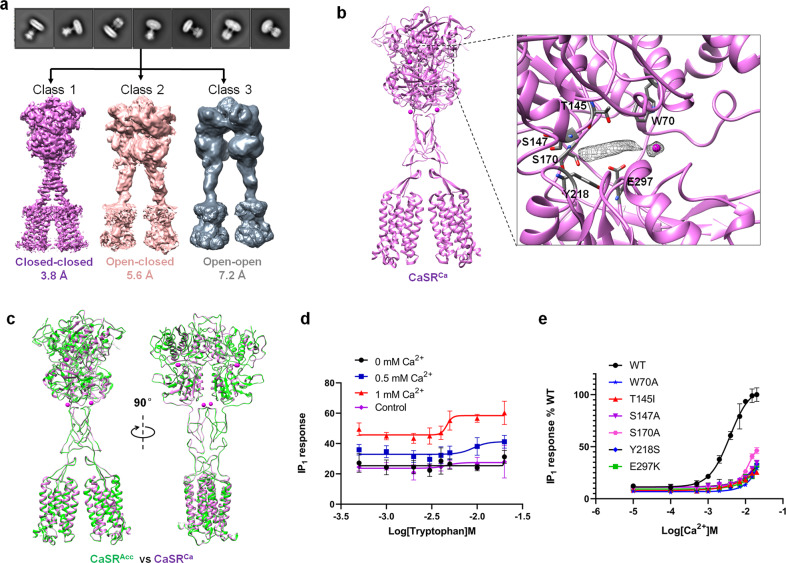
Cryo-EM structure of CaSR in the Ca^2+^-bound state. **a** Representative 2D class average images (upper panel) and three distinct classes of 3D reconstruction density maps are obtained with resolutions of 3.8 Å, 5.6 Å, and 7.3 Å, respectively (lower panel). **b** Cartoon representation of the dimeric Ca^2+^-bound CaSR structure in the closed-closed conformation (CaSR^Ca^). Extra density located in the cleft between LB1 and LB2 in the VFT domain is shown in mesh. The Ca^2+^ ion is shown as a magenta sphere. **c** Superposition of the overall structures of CaSR^Acc^ and CaSR in the Ca^2+^-bound closed-closed conformation. **d** L-Trp concentration-dependent activation of CaSR in the presence of Ca^2+^ ions. **e** Ca^2+^ concentration-dependent activation of CaSR mutants indicates that mutations of residues in L-Trp binding sites reduce receptor activation by Ca^2+^. The IP_1_ accumulation data in **d** and **e** represent the means ± SD of three independent experiments.

The structure of CaSR^Trp^ illustrates that the binding of L-Trp induces a conformational conversion of the VFT domain from open to closed, pushing CaSR into an intermediate active state. This raises an important question of whether Ca^2+^ ions alone can activate CaSR in the absence of L-Trp. To address this issue, we purified full-length CaSR in a buffer supplemented with 20 mM Ca^2+^ and applied the sample for cryo-EM. After extensive 3D classification, three different 3D models were obtained, with resolutions of 3.8 Å, 5.6 Å, and 7.3 Å, respectively (Supplementary information, Fig. S10). The VFT domains of the three models adopt closed-closed, open-closed, and open-open conformations, corresponding to the conformations of VFT domains in CaSR^Icc^, CaSR^Ioc^, and CaSR^Ioo^, respectively (Fig. 3a).

The 3.8 Å map showed clear extra densities in both VFT domains. The densities were located in the clefts between LB1 and LB2, overlapping with the location of L-Trp in the CaSR^Acc^ structure (Fig. 3b). This observation indicated that ambient amino acid (or amino acid derivative) could have bound to CaSR, just like that observed in the structure of CaSR^Icc^. We then built the structure model for the 3.8 Å map (designated as CaSR^Ca^) (Fig. 3b). Structural comparison demonstrated that the structure of CaSR^Ca^ is almost identical to that of CaSR^Acc^, representing the fully active state of CaSR (Fig. 3c). One reasonable explanation for this observation is that the ambient amino acid (or amino acid derivative) and supplemented Ca^2+^ jointly activate the CaSR receptor. In sharp contrast to CaSR^Ca^, the 5.6 Å and 7.3 Å maps show inactive conformations of CaSR, with the CRDs and TMDs separated (Fig. 3a).

Our functional data based on intracellular IP_1_ accumulation upon activation of CaSR showed that the activation of CaSR is dependent on L-Trp and Ca^2+^. In our experiments, the IP_1_ response was detected without exogenous Ca^2+^ ion supplementation. It is likely that endogenous Ca^2+^ ions and amino acids activate CaSR. In the presence of 0.5 mM or 1 mM exogenous Ca^2+^ ions, increased sensitivity to L-Trp was observed based on their ability to activate IP_1_ responses (Fig. 3d). More importantly, our data indicated that the residues involved in L-Trp binding (W70, T145, S147, S170, Y218, E297) are crucial for Ca^2+^-dependent receptor activation. The mutation of each of the above residues abolished Ca^2+^-induced receptor activation (Fig. 3e; Supplementary information, Fig. S11a and Table S2), which is consistent with previous functional studies of these residues.^4,25–27^ These findings suggest that the activation of CaSR requires the presence of Ca^2+^ and L-Trp simultaneously.

### Ca^2+^ induces a conformational transition of CaSR from CaSR^Icc^ to CaSR^Acc^

Cryo-EM structures revealed that CaSR^Icc^ and CaSR^Acc^ have different overall architectures, especially the CRDs and TMDs in the dimeric receptor. CaSR^Acc^ adopts a more compact architecture than CaSR^Icc^ (Supplementary information, Fig. S12). With the Cα atom of Asp234 taken as a reference, the LB2 in CaSR^Icc^ is separated by ~33.0 Å, and this distance is reduced to 17.8 Å in CaSR^Acc^. The CRDs of CaSR in both inactive and active states extend almost straight down from the C-terminus of the VFT domain towards the membrane. In CaSR^Icc^, Asp587 of the CRD is separated by 40.2 Å, whereas this distance is reduced to 8.9 Å in CaSR^Acc^. Moreover, the TMDs are completely separated in CaSR^Icc^. A distance of 17.0 Å is observed between the backbones of the TM6 helices of the two subunits, which are the closest pair of transmembrane helices. The TMDs of CaSR^Acc^ show a strong interaction along their TM6 helices, with a backbone separation distance of 5.7 Å at Ser827. As a result, extended interactions are observed between the VFT, CRD, and TMD of each subunit in the active CaSR structure.

**Fig. 4:**
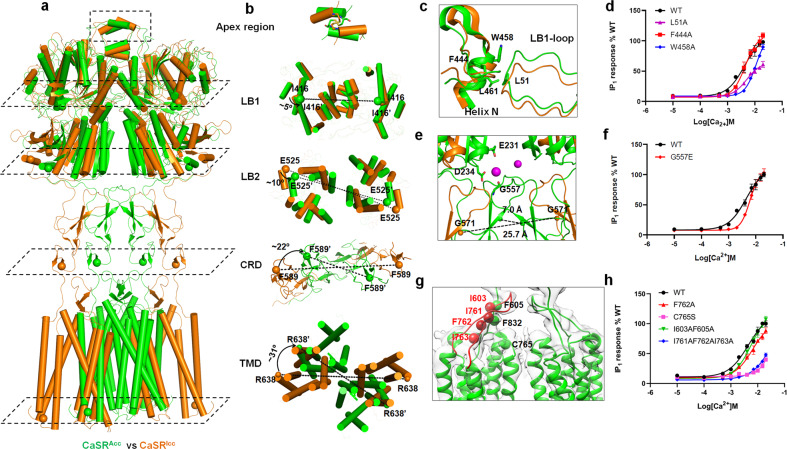
Conformational changes of CaSR upon Ca^2+^ binding. **a** Structural comparison of CaSR dimers in inactive (orange) and active (green) states. The dimeric CaSR structures are viewed in slices parallel to the membrane plane. **b** Gradual increase in the rotational angles of different subdomains down to the TMD along the symmetric axis of the inactive (orange) and active (green) CaSR dimers. The rotational angles of each subdomain are indicated. **c** Conformational changes of the interface between the LB1 loop and helix N. In Ca^2+^/L-Trp-bound active CaSR, the LB1 loop (green) reaches across the dimerization interface between two LB1 regions to contact helix N in the adjacent subunit, leading to domain rotation along the dimer axis. The LB1 loop and helix N in inactive CaSR are shown in orange. **d** Mutations of residues in hydrophobic sites in the LB1 loop (L51A) and helix N (F444A, W458A) reduced receptor activation by Ca^2+^. The IP_1_ accumulation data represent the means ± SD of three independent experiments. **e** Ca^2+^ ions were coordinated by D234, E231 in the LB2 region of one subunit, and G557 in the CRD of the other subunit, increasing the proximity of G557 residues of the two subunits in the active CaSR compared with that in the inactive CaSR. **f** The G557E mutation decreased the Ca^2+^-induced receptor response. The IP_1_ accumulation data represent the means ± SD of three independent experiments. **g** Structure model and cryo-EM map showing interactions between ECL2 of the TMD and the CRD. Critical residues at the CRD–TMD interface are shown as spheres at their Cα positions (I761, F762, I763, I603, and F605), indicating hydrophobic interactions between ECL2 and the linker region connecting the CRD and TMD. **h** Site mutations disrupting hydrophobic interactions between ECL2 and the CRD–TMD linker decreased the sensitivity of CaSR to Ca^2+^, as shown by Ca^2+^-stimulated IP_1_ accumulation assay. Data represent the means ± SD of three independent experiments.

Overall, superimposition of the CaSR^Acc^ and CaSR^Icc^ structures shows a gradual increase in the rotation angles of the LB1 and LB2 regions, CRDs, and finally TMDs of the two structures relative to the C2 axis of CaSR homodimer by ~5°, 10°, 22°, and 31°, respectively (Fig. 4a, b). This observation indicates that Ca^2+^ binding leads each individual domain of CaSR to twist. The twisting of CaSR allows the LB2, CRD, and TMD in the two subunits to move closer to each other, resulting in a more compact architecture of active CaSR compared to inactive CaSR.

### Conformational changes in the ECD between CaSR^Icc^ and CaSR^Acc^

Based on the structures of the inactive state and L-Trp-bound CaSR, L-Trp binding could induce the closed-closed conformation of the VFT domains, leading to conformational conversion from Ioo to Icc. A structural comparison of the VFT domains derived from CaSR^Icc^ and CaSR^Acc^ reveals only minor changes in the VFT domain upon agonist binding (Fig. 1f). The stabilized VFT domain thus could act as a rigid body during CaSR activation upon Ca^2+^ binding.

Ca^2+^-induced domain twisting in CaSR is thought to be regulated in part by a patch of hydrophobic residues formed by the B and C helices at the apical surface of the VFT domain. Interestingly, a long arm-like loop in the LB1 regions (LB1 loop) stretches from one subunit to its binding partner in both inactive and active CaSR structures. The LB1 loop contacts the helix in the LB1 region (helix N) of the other subunit through a hydrophobic interaction, which is mediated primarily by Leu51 in the loop and Phe444 and Trp458 in the helix (Fig. 4c; Supplementary information, Fig. S13). This hydrophobic interaction stabilizes the LB1 loop in an extended conformation, prompting the loop to reach across the dimerization interface in LB1 and facilitating consequent domain rotation along the dimer axis. Through the hydrophobic interaction between the LB1 loop and helix N, the conformational changes in these two regions could lead to twisting of the dimeric VFT domain and finally generate new interfaces in the active CaSR. The interaction between the LB1 loop and helix N of the adjacent subunit was found to be functionally important. The mutation of hydrophobic residues to alanine residues in the LB1 loop (L51A) and helix N (F444A, W458A) reduced receptor activation by Ca^2+^ (Fig. 4d; Supplementary information, Fig. S11b and Table S2). Therefore, this intersubunit domain interaction and conformational changes at the interface between LB1 regions are thought to initiate domain twisting in CaSR homodimer.

As a result of LB1 region twisting, the LB2 regions of the two subunits approach each other, expanding the homodimeric interactions involving the LB2 regions. Furthermore, the CRDs of the two subunits interact to form a large homodimeric interface unique to CaSR in the active state (Supplementary information, Fig. S4a, f). The CRDs are brought into close contact by motion involving the LB2 region since the two domains within each subunit are rigidly associated. The second Ca^2+^-binding site is located at the junction between the LB2 region and CRD and is involved in Ca^2+^ coordination by Glu231 and Asp234 in one subunit and Gly557 in the adjacent subunit^6^ (Fig. 4e). The G557E mutation led to decreased Ca^2+^ sensitivity of CaSR (Fig. 4f; Supplementary information, Fig. S11c and Table S2). Ca^2+^ binding in this site could compensate for the two negatively-charged residues (Glu231 and Asp234) and contribute to stabilizing the interaction between the two subunits of CaSR dimer induced by domain twisting.

### ECL2 relays the twist at the ECD to the TMD

Determining how conformational change is propagated from the ECD to the TMD upon agonist binding is crucial to understanding the mechanism of CaSR activation. In mGluR5, ECL2 in the TMD was identified as necessary to relay agonist-induced conformational changes to the TMD, as it provides a second, rigid attachment point between the ECD and TMD.^17^ Herein, our cryo-EM structure reveals an intra-subunit interaction between CRD and ECL2 in CaSR^Acc^.

Due to the local low resolution of the map, the side chains of the residues in the CRD and ECL2 regions of CaSR^Acc^ could not be assigned. However, the cryo-EM structure of CaSR^Acc^ clearly shows that residues Ile761, Phe762, and Ile763 in ECL2 and residues Ile603, Phe605, and Phe832 in the CRD–TM1 boundary are in close proximity (Fig. 4g). Therefore, we suggest that the CRD–ECL2 interface is maintained by hydrophobic interactions between these residues. The single-site mutant F762A, double-site mutant I603A/F605A and triple-site mutant I761A/F762A/I763A, which were designed to disrupt hydrophobic interactions, were observed to decrease the sensitivity of CaSR to Ca^2+^ in the IP_1_ accumulation assay (Fig. 4h; Supplementary information, Fig. S11d and Table S2). Moreover, Cys765 in ECL2 forms a disulfide bond with Cys677 in TM3, which could also contribute to relaying the twist at the ECD to the TMD. Our structural and functional data strongly imply that the hydrophobic interactions between ECL2 and CRD are important for domain twisting-mediated CaSR activation.

Notably, the apex region of ECL2 was found to be rich in acidic residues, including Glu755, Glu757, Asp758, and Glu759 (Supplementary information, Fig. S14a). Extra density in the density map could be observed at the interface between the negatively-charged tips of the two ECL2 regions, which most likely represents a cation (Supplementary information, Fig. S14b). We suspected that the cation is coordinated by Glu755-Leu756-Glu757 residues from the two subunits, stabilizes the interface between the negatively-charged ECL2 regions through charge neutralization, thus prompting the activation of CaSR. Alanine substitution of Glu755 and Glu757 resulted in increased ability of CaSR to activate IP_1_ responses (Supplementary information, Figs. S14c, S11e). These observations reveal that relieving the intersubunit electrostatic repulsion between the ECL2 regions could facilitate the activation of CaSR.

### Reorientation of the CaSR TMD during activation

**Fig. 5:**
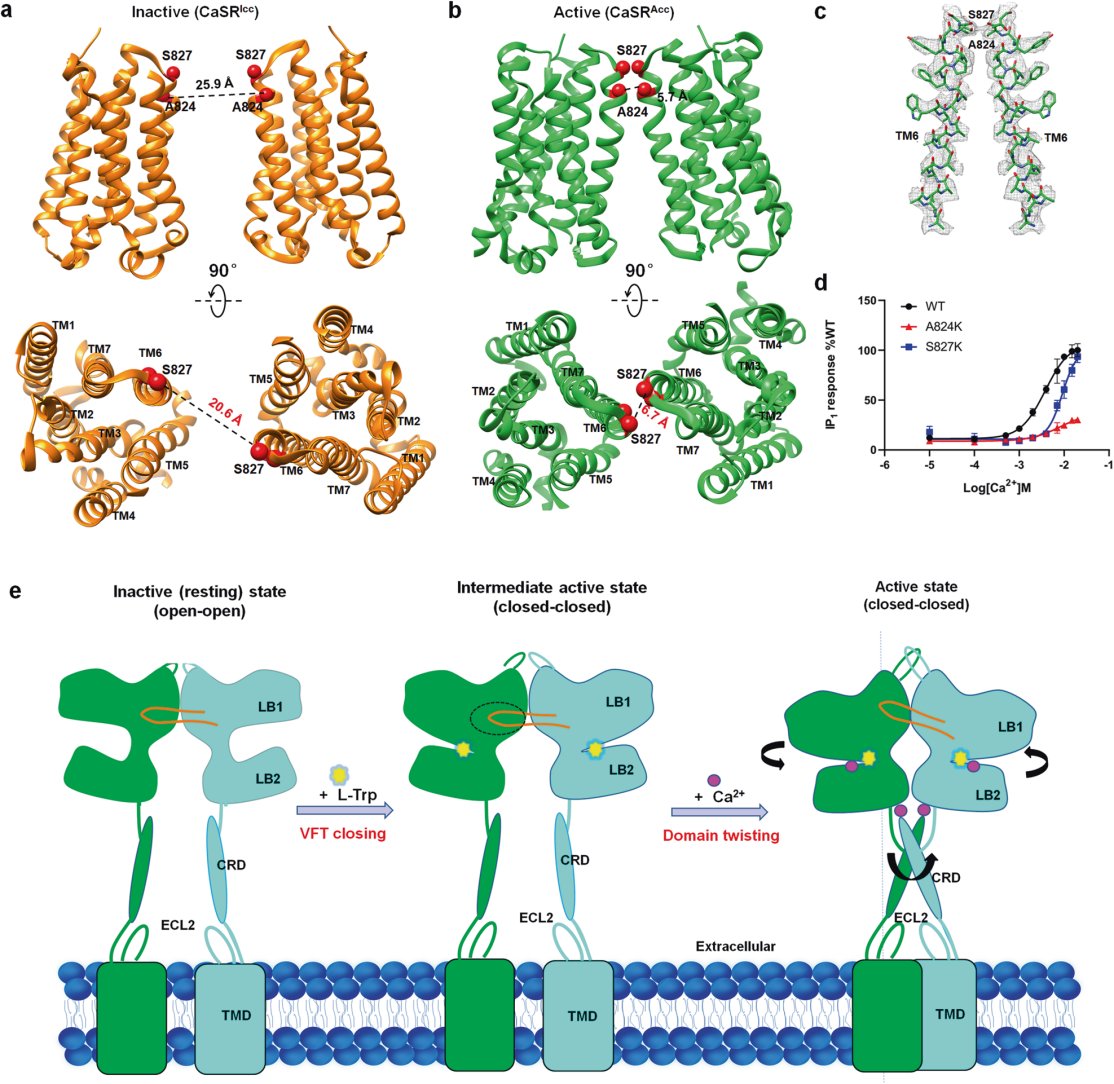
Rotation of the TMDs of CaSR during activation. **a**, **b** Compact dimerized TMDs from inactive CaSR (**a**) and active CaSR (**b**) are shown as cartoons in side view (upper panel) and top view (lower panel). A824 and S827, which are located in TM6, are indicated by red spheres. **c** Representative cryo-EM map and fitted atomic model of TM6 of CaSR^Acc^, indicating the agreement between the map and the model. **d** Mutations in residues contributing to TM6-mediated contact points (A824K and S827K) decreased the Ca^2+^-induced receptor response. The data represent the means ± SD of three independent experiments performed in triplicate. **e** A hypothetical model for cooperative activation of CaSR by L-Trp and Ca^2+^. The binding of the L-Trp molecule in the cleft of the VFT domain closes and stabilizes the VFT domain. Conformational changes in the LB1 loop region upon Ca^2+^ binding initiate twisting of the VFT domains. Ca^2+^ binds to the VFT domain and the junction between the LB2 region and the CRD, bringing the LB2 region and CRD closer through rigid-body domain twisting. The increased proximity of the LB2 regions is propagated to the TMDs through the interaction between the CRD and TMD-ECL2 region. A series of domain twisting motions between domains of CaSR dimer increase the proximity of the TMDs and produce a new TM6-mediated TMD interface for downstream signal transduction.

Recent cryo-EM structures of mGluR5 and GABA_B_ revealed that, in addition to moving closer to each other, each TMD rotates upon activation.^17,20,21^ In the CaSR structures reported here, we observed reorientation of the TMD similar to that observed in the mGluR5 and GABA_B_ structures (Fig. 5a, b; Supplementary information, Fig. S15). Upon agonist binding, the TMDs of the two CaSR subunits move closer together. Each TMD also rotates upon agonist binding. More importantly, the propagation of structural changes led to a TM6–TM6 interface that appears to be a hallmark of GPCR activation, as this interface was observed in mGluR5 and confirmed by cysteine-mediated crosslinking at the extracellular face of TM6 in mGluR5.^17^ In CaSR, mutants A824K and S827K in the TMD region were prepared for functional analysis, showing marked defects in activating IP_1_ responses (Fig. 5c, d; Supplementary information, Fig. S11f and Table S2). The A824K mutation mentioned above has been reported to be disease related,^7^ indicating that the TM6–TM6 interface in active CaSR is critical to the function of the receptor.

## Discussion

Herein, we report the cryo-EM structures of full-length human CaSR in inactive and active states. Combined with functional assays, these structures enable us to propose a structural framework for CaSR activation (Fig. 5e). In the ligand-free resting state, the VFT domain of CaSR could adopt an open conformation, consistent with that observed in mGluR5 or GABA_B_ in a resting state.^17,20–23^ The binding of L-Trp at the LB1–LB2 cleft induces the closure of VFT and stabilizes VFT in a closed conformation, resulting in a unique intermediate closed-closed state of CaSR during activation. In the presence of a high concentration of Ca^2+^ ions, Ca^2+^ binding could induce slight conformational changes in the LB1–LB1 interfaces, initiating domain twisting in the two VFT domains. Binding of a Ca^2+^ ion at the LB2–CRD interface compensates for repulsive interactions between negatively-charged residues (Glu231, Asp234) at this junction, facilitating the proximity between the LB2 regions and leading them to twist. The increased proximity and twisting of LB2 regions are relayed to further increase the proximity and twisting of the CRD domains. The intra-subunit hydrophobic interactions between ECL2 and CRD, together with a potentially Ca^2+^-mediated intersubunit interaction between the ECL2 regions, play a vital role in transducing rigid-body twisting motions in the ECD to the TMD, increasing the proximity of the TMDs. The TMDs of the two CaSR subunits reorient to form a new TM6-mediated interface that is signaling competent. Similar rearrangements have been observed in other class C GPCRs, including mGluR5 and GABA_B_,^17,20,21^ suggesting that a conformational transition by which the TMDs come into close proximity is a hallmark of the activation of members of this family.^17^

Previous crystal structures of the extracellular domains of CaSR, GABA_B_, and the mGlu receptors,^4,13,19,28,29^ as well as cryo-EM structures of full-length mGluR5 and GABA_B_, have suggested that agonists induce large conformational changes and closure of the VFT domain during the activation of the class C GPCR family members.^16,17,19–21^ Thus, closure of the VFT domain upon agonist binding was thought to initiate the activation of dimeric class C GPCRs and be a structural mechanism shared by all class C GPCRs during their ligand-dependent activation.^16,17,20,21,30–32^ Notably, our work clarifies that L-Trp is not able to activate CaSR alone. L-Trp binding induces and stabilizes the closed conformation of the VFT domain, pushing CaSR to an intermediate active state, which is a crucial step in activating the receptor. However, L-Trp is not able to induce TMD rotation and thus not able to activate the receptor. Ca^2+^ binding initiates and relays the twist at the VFT domains to the TMDs, consequently inducing close contact between the two TMDs of dimeric CaSR and activating the receptor. Taken together, our findings demonstrate that amino acids and calcium ions act jointly to fully activate CaSR.

Malfunction of CaSR due to either its own mutation or disordered calcium homeostasis has been implicated in several human diseases.^7,15,33,34^ Allosteric modulation of CaSR has been actively studied as a means to fight secondary hyperparathyroidism (SHPT) caused by defective calcium and phosphorus homeostasis.^15,35–38^ Both small-molecule (e.g., cinacalcet) and peptidic (e.g., etelcalcetide) activators of CaSR have been developed as calcimimetic drugs for SHPT, but their functional mechanisms remain to be elucidated.^12,39–42^ Structural insights into CaSR activation may provide new knowledge that is useful for the development of drugs with tailored properties and fewer side effects. For instance, comparison of the resting and active structures of CaSR identified extended interfaces between the two LB2 regions, CRDs, ECL2 regions, and TMDs. These activation-dependent interfaces may present interesting opportunities for therapeutic intervention and allow broadening of the chemical spectrum of CaSR ligands using structure-based drug design. Of particular interest is an observed long stretch of negatively-charged residues at the interface between the LB2 regions, the CRDs, and the ECL2 regions of each subunit. In active mGluR5, a distribution of discrete charges was observed in the same region (Supplementary information, Fig. S16). This CaSR interface provides a topographically distinct binding pocket for positively-charged allosteric modulators that may host an interaction with the recently developed D-peptide etelcalcetide, which bears four D-arginine residues.^41^ Thus, the distinctive activation mode of CaSR uncovered in this work may open new modular design strategies, differing from the knowledge gained from other class C GPCR family members, including mGluR5 and GABA_B_.

## Methods and materials

### Cloning and protein expression

The DNA sequence of full-length human CaSR (UniProt: P41180) was synthesized with optimized codons by Sangon Biotech and then cloned into the pFastBac1 vector (Invitrogen). The original signal sequence (1–19 aa) of CaSR was substituted by that of haemagglutinin (HA), followed by a Flag epitope and a TEV cleavage site. Octahistidine tag was also added at the C-terminus of CaSR. Bac-to-Bac Baculovirus Expression System (Invitrogen) was used to generate high-titer recombinant baculovirus. *Spodoptera frugiperda* (Sf9) cells (Invitrogen) at a density of 2.5 × 10^6^ cells/mL were infected by viral stock at an MOI (multiplicity of infection) of 3. The baculovirus-transfected Sf9 cells were cultured at 27 °C for 60 h, collected by centrifugation, and stored at –80 °C until use.

### Protein purification of CaSR

To prepare inactive form CaSR, cells were collected and solubilized in hypotonic buffer consisting of 20 mM HEPES (pH 7.5), 20 mM NaCl, 10 mM EDTA, 1.5% (w/v) DDM, 0.3% (w/v) CHS and cocktail protease inhibitor (Roche). The supernatant fraction isolated by centrifugation at 170,000× *g* for 45 min was collected and incubated with Anti-Flag G1 Affinity Gel (GenScript) for 1 h at 4 °C. Protein-bound gel was washed with a washing buffer consisting of 20 mM HEPES (pH 7.5), 150 mM NaCl, 5 mM EDTA, 0.1% (w/v) DDM and 0.02% (w/v) CHS, and then eluted in an elution buffer consisting of 20 mM HEPES (pH 7.5), 150 mM NaCl, 5 mM EDTA, 0.1% (w/v) DDM, 0.02% (w/v) CHS and 200 μg/mL FLAG peptide. The eluted protein was concentrated and further purified by size-exclusion chromatography on Superpose 6 increase column (GE Healthcare) in 20 mM HEPES (pH 7.5), 150 mM NaCl, 5 mM EDTA, 0.01% (w/v) DDM and 0.002% (w/v) CHS. Finally, inactive CaSR was concentrated to ~5 mg/mL for single-particle cryo-EM sample preparation.

To prepare the CaSR–Ca^2+^/Trp sample, collected cells were lysed and solubilized in hypotonic buffer consisting of 20 mM HEPES (pH 7.5), 10 mM MgCl_2_, 20 mM KCl, 1.5% (w/v) DDM, 0.3% (w/v) CHS and cocktail protease inhibitor. The supernatant fraction was isolated and incubated with Anti-Flag G1 Affinity Gel (GenScript) for 1 h at 4 °C. Protein-bound gel was washed with buffer consisting of 20 mM HEPES (pH 7.5), 150 mM NaCl, 0.1% (w/v) DDM and 0.02% (w/v) CHS, and then eluted in buffer comprised of 20 mM HEPES (pH 7.5), 150 mM NaCl, 200 μg/mL FLAG peptide, 0.1% (w/v) DDM and 0.02% (w/v) CHS. The eluted protein was further purified by size-exclusion chromatography on a Superpose 6 increase column (GE Healthcare) in 20 mM HEPES (pH 7.5), 150 mM NaCl, 0.01% (w/v) DDM and 0.002% (w/v) CHS. Monomeric peak fractions of CaSR protein was concentrated to ~5 mg/mL, and a final concentration of 10 mM L-Trp and 10 mM CaCl_2_ were added into protein solution.

To prepare CaSR–Trp sample, cells were collected and solubilized in hypotonic buffer consisting of 20 mM HEPES (pH 7.5), 20 mM NaCl, 10 mM EGTA, 1.5% (w/v) DDM, 0.3% (w/v) CHS and cocktail protease inhibitor. The supernatant fraction isolated by centrifugation at 170,000× *g* for 45 min was collected and incubated with Anti-Flag G1 Affinity Gel (GenScript) for 1 h at 4 °C. Protein-bound gel was washed with a washing buffer consisting of 20 mM HEPES (pH 7.5), 150 mM NaCl, 5 mM EGTA, 0.1% (w/v) DDM and 0.02% (w/v) CHS, and then eluted in an elution buffer consisting of 20 mM HEPES (pH 7.5), 150 mM NaCl, 5 mM EGTA, 0.1% (w/v) DDM, 0.02% (w/v) CHS and 200 μg/mL FLAG peptide. The eluted protein was concentrated and further purified by size-exclusion chromatography on Superpose 6 increase column (GE Healthcare) in 20 mM HEPES (pH 7.5), 150 mM NaCl, 5 mM EGTA, 0.01% (w/v) DDM and 0.002% (w/v) CHS. The peak fraction of CaSR protein was collected and supplemented with 10 mM Trp. Finally, CaSR–Trp was concentrated to ~5 mg/mL for single-particle cryo-EM sample preparation.

To prepare CaSR–Ca^2+^ sample, cells were collected and solubilized in hypotonic buffer consisting of 20 mM HEPES (pH 7.5), 20 mM NaCl, 10 mM EGTA, 1.5% (w/v) DDM, 0.3% (w/v) CHS and cocktail protease inhibitor. The supernatant fraction isolated by centrifugation at 170,000× *g* for 45 min was collected and incubated with Anti-Flag G1 Affinity Gel (GenScript) for 1 h at 4 °C. Protein-bound gel was washed with a washing buffer consisting of 20 mM HEPES (pH 7.5), 150 mM NaCl, 20 mM CaCl_2_, 0.1% (w/v) DDM and 0.02% (w/v) CHS, and then eluted in an elution buffer consisting of 20 mM HEPES (pH 7.5), 150 mM NaCl, 20 mM CaCl_2_, 0.1% (w/v) DDM, 0.02% (w/v) CHS and 200 μg/mL FLAG peptide. The eluted protein was concentrated and further purified by size-exclusion chromatography on Superpose 6 increase column (GE Healthcare) in 20 mM HEPES (pH 7.5), 150 mM NaCl, 20 mM CaCl_2_, 0.01% (w/v) DDM and 0.002% (w/v) CHS. Finally, CaSR protein was concentrated to ~5 mg/mL for single-particle cryo-EM sample preparation.

### Single-particle cryo-EM sample preparation and data acquisition

An aliquot of 3 μL purified inactive CaSR, CaSR–Ca^2+^/Trp, CaSR–Trp or CaSR–Ca^2+^ sample was applied to plasma-treated (H_2_/O_2_, 10 s) holey carbon grids (GryoMatrix-M024, R1.2/1.3, 300 mesh, Au), at a concentration of ~5 mg/mL and subsequently vitrified using a Vitrobot Mark IV (Thermo Fischer Scientific), blotted for 6 s at 100 % humidity and 4 °C, and then plunge frozen into liquid ethane cooled by liquid nitrogen. Using software SeiralEM,^43^ cryo-EM images of CaSR–Ca^2+^/Trp (8624 stacks), CaSR–Ca^2+^ (12,030 stacks), and inactive CaSR dataset 1 images (13,420 stacks) were collected on a Titan Krios operated at 300 kV at a nominal magnification of 29,000× using a Gatan K2 Summit direct detection camera in counting mode, corresponding to a pixel size of 1.014 Å. Image stacks were obtained with a dose rate of 8 e^–^/pixel/s and total exposure time of 8 s with 0.2 s per frame, resulting in a total dose of 62 electrons per Å^2^. The defocus range was set to –1.3 to –2.5 μm. The dataset 2 of inactive CaSR (5238 stacks) were collected on a Titan Krios operated at 300 kV at a nominal magnification of 22,500× using a Gatan K3 camera, in super-resolution mode with pixel size of 0.53 Å and a defocus range of –1.3 to –2.5 μm. Image stacks were obtained with a dose rate of 16.85 e^–^/pixel/s and total exposure time of 4 s with 0.1 s per frame, resulting in a total dose of 60 electron per Å^2^. The CaSR–Trp datasets (6133 stacks) were collected on a Titan Krios operated at 300 kV at a nominal magnification of 29,000× using a Gatan K2 camera, in counting mode with pixel size of 1.01 Å and a defocus range of –1.3 to –2.2 μm. Image stacks were obtained with a dose rate of 10 e^–^/pixel/s and total exposure time of 5.76 s with 0.16 s per frame, resulting in a total dose of 56.5 electron per Å^2^.

### Cryo-EM image processing

Dose-fractionated image stacks were subjected to beam-induced motion correction and dose-weighting using UCSF MotionCor2.^44^ Contrast transfer function parameters were estimated with Gctf.^45^ Around 3000 particles were manually picked and processed with reference-free 2D classification in RELION to generate templates for auto-picking using RELION or Gautomatch (written by Kai Zhang).

For the inactive CaSR dataset, 3,444,116 particles were auto-picked from K2 images and 2,185,931 particles were auto-picked from K3 images. Auto-picked particles were cleaned up with 2 rounds of 2D classification separately in Relion-3.1.^46^ Particles from well-defined 2D averages were selected and joined together for 3D classification without symmetry. A 3D initial model de novo from the 2D average particles was generated using stochastic gradient descent (SGD) algorithm in Relion. The 60 Å low-pass filtered initial model was used as a template for 3D classification into four classes. Class 1 and class 3 from 3D classification results (Supplementary information, Fig. S2) were selected and re-extracted into the original pixel size. After 3D refinement with C2 symmetry, particle polishing, and CTF refinement, the resulting 3D reconstructions of class 1 from 255,096 particles and class 3 from 210,908 particles yielded EM maps with resolutions of 4.5 Å and 6.8 Å, respectively. Class 2 from 3D classification results was selected and re-extracted into the original pixel size. After 3D refinement with C1 symmetry, particle polishing, and CTF refinement, the resulting 3D reconstructions of class 2 from 233,923 particles yielded an EM map with a resolution of 5.7 Å.

For CaSR–Ca^2+^/Trp, 1,382,094 particles were automatically picked and extracted from 8624 micrographs. The extracted particles were binned 4 times and subjected to a 2D classification, and a total of 528,063 particles were selected and binned 2 times for 3D classifications. 3D initial model was generated de novo from the 2D average particles using SGD algorithm in Relion. One of the 3D classes showed good secondary structural features, and their particles were selected and re-extracted into the original pixel size of 1.014 Å. After 3D refinement with C2 symmetry, particle polishing and CTF refinement, the resulting 3D reconstructions from 229,926 particles yielded an EM map with a resolution of 3.5 Å.

For CaSR–Trp, 2,169,546 particles were automatically picked and extracted from 6133 micrographs. The extracted particles were binned 4 times and subjected to a 2D classification, and a total of 856,546 particles were selected and binned 2 times for 3D classifications. 3D initial model was generated de novo from the 2D average particles using SGD algorithm in Relion. One of the 3D classes showed good secondary structural features, and their particles were selected and re-extracted into the original pixel size of 1.01 Å. After 3D refinement with C2 symmetry, particle polishing, and CTF refinement, the resulting 3D reconstructions from 240,292 particles yielded an EM map with a resolution of 4.4 Å.

For the CaSR–Ca^2+^ dataset, 3,701,279 particles were auto-picked from 12,030 images, and 2,185,931 particles were auto-picked from K3 images. The extracted particles were binned 4 times and subjected to a 2D classification. A total of 1,240,840 particles from well-defined 2D averages were selected and binned 2 times for 3D classification without symmetry. After two-round 3D classification, particles of classes 1, 2, and 3 were selected and re-extracted into the original pixel size. After 3D refinement with C1 symmetry, particle polishing, and CTF refinement, the resulting 3D reconstructions of class 1 from 246,468 particles yielded an EM map with a resolution of 5.6 Å. After 3D refinement with C2 symmetry, particle polishing, and CTF refinement, the resulting 3D reconstructions of class 2 from 199,309 particles and class 3 from 315, 050 particles yielded EM maps with resolutions of 7.2 Å and 3.8 Å, respectively.

The resolutions were estimated by applying a soft mask around the protein density and the gold-standard Fourier shell correlation (FSC) = 0.143 criterion. Local resolution was determined using ResMap^47^ with unfiltered half-reconstructions as input maps.

### Model building and refinement

First, we built and refined the active state CaSR model. Initially, the crystal structure of active form CaSR-ECD (PDB code: 5k5s)^48^ was fitted into the 3D EM map of active state CaSR using UCSF Chimera.^49^ Every residue was manually examined in Coot.^50^ The TMD model of active state CaSR was derived using the mGluR5 (PDB code: 6n51)^17^ structure as a reference. The coordinates of mGluR5-TMD were fitted into the EM map by Chimera, and then the sequence of mGluR5-TMD were mutated to the corresponding residues in CaSR in Coot. The initial model was subjected to iterative manual rebuilding in Coot and real-space refinement in PHENIX.^51^ The final structure was validated using the module “comprehensive validation (cryo-EM)” in PHENIX.^52^ The residues Gly363–Ala390, Arg638–Arg648, Val702–Leu723 and the C-terminal residues after Ile859 were not built due to the lack of corresponding densities. *N*-acetylglucosamine moieties were built to linking to Asn261, Asn287, Asn446, Asn468, Asn488, and Asn541 sites, respectively, based on the corresponding densities. Side chains of apex region (residues Asp121–His134) were removed due to the weak electron density corresponding to this segment. Side chains of most residues in the TMD were removed, except for Trp675, Arg752, Phe762, Trp818, Phe821, Pro823–Leu842, and Phe846, due to the weak electron density corresponding to this segment. The Cys677/Cys765 disulfide bond was built based on the corresponding bond (Cys644/Cys733) in mGluR5. To build the model of Ca^2+^-bound closed-closed state CaSR, the structure of active state CaSR was fitted into the EM map by Chimera, and then the initial model was subjected to iterative manual rebuilding in Coot and real-space refinement in PHENIX. To build the models of Trp-bound state CaSR and Icc state CaSR, the separated LB1 (Tyr20–Asn189 and Lys323–Gly487), LB2 (Asp190–Leu322 and Asn488–Asn541), CRD (Cys542–Ile599), and TMD (Ala600–Ile859) of active state CaSR structure were docked into the EM map independently and adjusted manually in Coot. Structure refinement and model validation were performed using PHENIX.

For the rest of four models fitting into their low-resolution maps, including the Ioc state, Ioo state, Ca^2+^-bound open-closed state, and Ca^2+^-bound open-open state CaSR, the separated LB1 and LB2 derived from active state CaSR structure were fitted as independent rigid bodies into those maps in Chimera, respectively. The residues in the junctions of LB1 and LB2 were manually examined. All the figures were generated using Chimera and PyMOL.

### IP_1_ accumulation assay

HEK-293T cells were seeded in six-well plates 24 h before transfection at a density of 3.5 × 10^5^ cells in 2 mL growth medium and transiently transfected with either the wild-type CaSR or mutants for 24 h. On the day of transfection, 3 µg DNA encoding each of the receptor constructs was incubated with 3.5 µL lipofectamine 3000 (Invitrogen™, L3000001) in a total of 75 µL OptiMEM (Gibco, 31985088) for 25 min at room temperature and then added to the cells. Culture medium was removed at 24 h post transfection, and the cells were washed with 1 mL DPBS (without Ca^2+^ and Mg^2+^; Gibco, 14190) immediately. Enzyme-free cell dissociation buffer (Sigma-Aldrich, C5914) was added into the six-well plates and incubated for 2 min. The cells were then resuspended with 1 mL stimulation buffer (10 mM HEPES, pH 7.4, 4.2 mM KCl, 146 mM NaCl, 50 mM LiCl, and 5.5 mM glucose). About 10,000 cells were added into 384-well plates (HTRF 384 well low volume plate, Cisbio) and stimulated with increasing concentrations of Ca^2+^ (Ca^2+^ concentrations were set as 0.01, 0.1, 0.5, 1.0, 2.0, 4.0, 7.0, 10.0, 15.0, and 20 mM, higher concentrations were avoided to protect the normal cell state) in stimulation buffer at 37 °C for 1 h. For concentration-dependent effects of Trp on CaSR activation, ~10,000 cells were added into 384-well plates and stimulated with increasing concentrations of Trp (Trp concentrations were set as 0.5, 1.0, 2.0, 3.0, 4.0, 5.0, 10.0, and 20 mM) in stimulation buffer at 37 °C for 1 h accompanied by 0, 0.5, 1.0 mM Ca^2+^, respectively. The reaction mixture was then added with an IP_1_ analog coupled to a D2 fluorophore (acceptor) and an anti-IP_1_-Cryptate (donor) (62IPAPEB, Cisbio). Native IP_1_ produced by cells resulting from activation of CaSR or mutants competed with D2-labeled IP_1_.^4,53^ Plates were read on a CLARIOstar microplate reader (BMG Labtech) 1 h later with excitation at 320 nm. The FRET signal was acquired with fluorescence ratio (665 nm/620 nm).^54^ Data analysis was performed using the non-linear regression algorithms (four-parameter logistic curve) in Prism (GraphPad Software, USA). Data points represent three independent experiments performed in triplicate. Statistics for mutational studies were performed using one-way analysis of variance followed by Dunnett’s test with wild-type receptors as the control.

### Cell surface expression level analysis

Cell surface expression was determined by flow cytometry. HEK-293T cells were seeded in six-well plates 24 h before transfection at a density of 3.5 × 10^5^ cells in 2 mL growth medium and transiently transfected with either the wild-type CaSR or mutants for 24 h. After transfection, cells were washed twice with 500 µL DPBS (without Ca^2+^ and Mg^2+^; Gibco, 14190) containing 3% BSA. The cells were then pelleted and resuspended in 100 µL DPBS containing 3% BSA and 1 µL anti-CaSR-antibody (5C10, ADD, ab19347). After incubation for at least 45 min at room temperature, the cells were washed 3 times by centrifugation at 400× *g* for 5 min and resuspended in ice-cold DPBS containing 3% BSA and PE-conjugated secondary antibody (IgG H&L, Phycoerythrin, ab7003). After incubation at 4 °C for 1 h in the dark, the cells were washed twice with DPBS and passed through a cell strainer, and finally cell surface expression was determined by flow cytometry on an BD FACS melody (BD Biosciences) by quantifying the PE-fluorescence (excitation at 488 nm, emission at 576 nm) when gating on the live cell population using forward and side scatter. Expression levels as measured by mean fluorescence were normalized to the expression level of wild-type CaSR. Each construct was performed in three independent experiments. Statistics for mutational studies were performed using one-way analysis of variance followed by Dunnett’s test with wild-type receptors as the control.

## Supplementary information

Supplementary information, Figure S1

Supplementary information, Figure S2

Supplementary information, Figure S3

Supplementary information, Figure S4

Supplementary information, Figure S5

Supplementary information, Figure S6

Supplementary information, Figure S7

Supplementary information, Figure S8

Supplementary information, Figure S9

Supplementary information, Figure S10

Supplementary information, Figure S11

Supplementary information, Figure S12

Supplementary information, Figure S13

Supplementary information, Figure S14

Supplementary information, Figure S15

Supplementary information, Figure S16

Supplementary information, Table S1

Supplementary information, Table S2

## Data Availability

Cryo-EM maps have been deposited in the Electron Microscopy Data Bank under accession codes: EMDB-30853, EMDB-30854, EMDB-30855, EMDB-30856, EMDB-30857 and EMDB-30858. The atomic coordinates have been deposited in the Protein Data Bank under accession codes: 7DTT, 7DTU, 7DTV, 7DTW.

## References

[1] Brown EM (1993). Cloning and characterization of an extracellular Ca(2+)-sensing receptor from bovine parathyroid. Nature.

[2] Hofer AM, Brown EM (2003). Extracellular calcium sensing and signalling. Nat. Rev. Mol. Cell Biol..

[3] Magno AL, Ward BK, Ratajczak T (2011). The calcium-sensing receptor: a molecular perspective. Endocr. Rev..

[4] Geng Y (2016). Structural mechanism of ligand activation in human calcium-sensing receptor. Elife.

[5] Saidak Z, Brazier M, Kamel S, Mentaverri R (2009). Agonists and allosteric modulators of the calcium-sensing receptor and their therapeutic applications. Mol. Pharmacol..

[6] Conigrave AD, Quinn SJ, Brown EM (2000). L-amino acid sensing by the extracellular Ca2+-sensing receptor. Proc. Natl. Acad. Sci. USA.

[7] Hendy GN, Guarnieri V, Canaff L (2009). Calcium-sensing receptor and associated diseases. Prog. Mol. Biol. Trans..

[8] Pidasheva S, D’Souza-Li L, Canaff L, Cole DE, Hendy GN (2004). CASRdb: calcium-sensing receptor locus-specific database for mutations causing familial (benign) hypocalciuric hypercalcemia, neonatal severe hyperparathyroidism, and autosomal dominant hypocalcemia. Hum. Mutat..

[9] Bai M, Trivedi S, Brown EM (1998). Dimerization of the extracellular calcium-sensing receptor (CaR) on the cell surface of CaR-transfected HEK293 cells. J. Biol. Chem..

[10] Pidasheva S (2006). Calcium-sensing receptor dimerizes in the endoplasmic reticulum: biochemical and biophysical characterization of CASR mutants retained intracellularly. Hum. Mol. Genet..

[11] Zhang C (2016). Structural basis for regulation of human calcium-sensing receptor by magnesium ions and an unexpected tryptophan derivative co-agonist. Sci. Adv..

[12] Keller AN (2018). Identification of global and ligand-specific calcium sensing receptor activation mechanisms. Mol. Pharmacol..

[13] Zhang C (2016). Molecular basis of the extracellular ligands mediated signaling by the calcium sensing receptor. Front. Physiol.

[14] Liu H (2020). Illuminating the allosteric modulation of the calcium-sensing receptor. Proc. Natl. Acad. Sci. USA.

[15] Hannan FM, Kallay E, Chang W, Brandi ML, Thakker RV (2018). The calcium-sensing receptor in physiology and in calcitropic and noncalcitropic diseases. Nat. Rev. Endocrinol..

[16] Kunishima N (2000). Structural basis of glutamate recognition by a dimeric metabotropic glutamate receptor. Nature.

[17] Koehl A (2019). Structural insights into the activation of metabotropic glutamate receptors. Nature.

[18] Geng Y (2012). Structure and functional interaction of the extracellular domain of human GABA(B) receptor GBR2. Nat. Neurosci..

[19] Geng Y, Bush M, Mosyak L, Wang F, Fan QR (2013). Structural mechanism of ligand activation in human GABA(B) receptor. Nature.

[20] Mao C (2020). Cryo-EM structures of inactive and active GABA_B_ receptor. Cell Res..

[21] Shaye H (2020). Structural basis of the activation of a metabotropic GABA receptor. Nature.

[22] Park J (2020). Structure of human GABA_B_ receptor in an inactive state. Nature.

[23] Papasergi-Scott MM (2020). Structures of metabotropic GABA_B_ receptor. Nature.

[24] Jiang, H. Cryo-EM structure determination captures new chemical modification of protein. *Sci. China Life Sci.*10.1007/s11427-021-1886-8 (2021). 10.1007/s11427-021-1886-8 33471275

[25] Zhang C (2014). Identification of an L-phenylalanine binding site enhancing the cooperative responses of the calcium-sensing receptor to calcium. J. Biol. Chem..

[26] Mun HC (2004). The venus fly trap domain of the extracellular Ca^2+^-sensing receptor is required for L-amino acid sensing. J. Biol. Chem..

[27] Zhang Z (2002). Three adjacent serines in the extracellular domains of the CaR are required for L-amino acid-mediated potentiation of receptor function. J. Biol. Chem..

[28] Muto T, Tsuchiya D, Morikawa K, Jingami H (2007). Structures of the extracellular regions of the group II/III metabotropic glutamate receptors. Proc. Natl. Acad. Sci. USA.

[29] Wu H (2014). Structure of a class C GPCR metabotropic glutamate receptor 1 bound to an allosteric modulator. Science.

[30] Frangaj A, Fan QR (2018). Structural biology of GABA_B_ receptor. Neuropharmacology.

[31] Kniazeff J (2004). Closed state of both binding domains of homodimeric mGlu receptors is required for full activity. Nat. Struct. Mol. Biol..

[32] Tsuchiya D, Kunishima N, Kamiya N, Jingami H, Morikawa K (2002). Structural views of the ligand-binding cores of a metabotropic glutamate receptor complexed with an antagonist and both glutamate and Gd3. Proc. Natl. Acad. Sci. USA.

[33] Pollak MR (1993). Mutations in the human Ca(2+)-sensing receptor gene cause familial hypocalciuric hypercalcemia and neonatal severe hyperparathyroidism. Cell.

[34] Ward BK, Magno AL, Walsh JP, Ratajczak T (2012). The role of the calcium-sensing receptor in human disease. Clin. Biochem..

[35] Brown EM (2010). Clinical utility of calcimimetics targeting the extracellular calcium-sensing receptor (CaSR). Biochem. Pharmacol..

[36] Egbuna OI, Brown EM (2008). Hypercalcaemic and hypocalcaemic conditions due to calcium-sensing receptor mutations. Best Pract. Res. Clin. Rheumatol..

[37] Leach K, Gregory KJ (2017). Molecular insights into allosteric modulation of Class C G protein-coupled receptors. Pharmacol. Res..

[38] Centeno PP (2019). Phosphate acts directly on the calcium-sensing receptor to stimulate parathyroid hormone secretion. Nat. Commun..

[39] Bushinsky DA (2015). Treatment of secondary hyperparathyroidism: results of a phase 2 trial evaluating an intravenous peptide agonist of the calcium-sensing receptor. Am. J. Nephrol..

[40] Hamano N, Komaba H, Fukagawa M (2017). Etelcalcetide for the treatment of secondary hyperparathyroidism. Expert Opin. Pharmacother..

[41] Harada K (2019). Pharmacology of Parsabiv((R)) (etelcalcetide, ONO-5163/AMG 416), a novel allosteric modulator of the calcium-sensing receptor, for secondary hyperparathyroidism in hemodialysis patients. Eur. J. Pharmacol..

[42] Nemeth EF (2004). Pharmacodynamics of the type II calcimimetic compound cinacalcet HCl. J. Pharmacol. Exp. Ther..

[43] Mastronarde DN (2003). SerialEM: a program for automated tilt series acquisition on tecnai microscopes using prediction of specimen position. Microsc. Microanal.

[44] Zheng SQ (2017). MotionCor2: anisotropic correction of beam-induced motion for improved cryo-electron microscopy. Nat. Methods.

[45] Zhang K (2016). Gctf: Real-time CTF determination and correction. J. Struct. Biol..

[46] Zivanov J (2018). New tools for automated high-resolution cryo-EM structure determination in RELION-3. Elife.

[47] Kucukelbir A, Sigworth FJ, Tagare HD (2014). Quantifying the local resolution of cryo-EM density maps. Nat. Methods.

[48] Yoder N, Yoshioka C, Gouaux E (2018). Gating mechanisms of acid-sensing ion channels. Nature.

[49] Pettersen EF (2004). UCSF Chimera–a visualization system for exploratory research and analysis. J. Comput. Chem.

[50] Emsley P, Lohkamp B, Scott WG, Cowtan K (2010). Features and development of Coot. Acta Crystallogr. D Biol. Crystallogr..

[51] Adams PD (2010). PHENIX: a comprehensive Python-based system for macromolecular structure solution. Acta Crystallogr. D Biol. Crystallogr..

[52] Afonine PV (2018). New tools for the analysis and validation of cryo-EM maps and atomic models. Acta Crystallogr. D Struct. Biol..

[53] Dore AS (2014). Structure of class C GPCR metabotropic glutamate receptor 5 transmembrane domain. Nature.

[54] Norskov-Lauritsen L, Thomsen AR, Brauner-Osborne H (2014). G protein-coupled receptor signaling analysis using homogenous time-resolved Forster resonance energy transfer (HTRF(R)) technology. Int. J. Mol. Sci..

