# Supplementary material for: Structural mechanism of cooperative activation of the human calcium-sensing receptor by Ca2+ ions and L-tryptophan
Source: Cell Res. 2021 Feb 18;31(4):383–94. doi: 10.1038/s41422-021-00474-0 (PMC8115157; doi:10.1038/s41422-021-00474-0)
Supplement: Supplementary file 1 — Supplementary information, Figure S1 [file 41422_2021_474_MOESM1_ESM.pdf]

Supplementary information, Figure S1

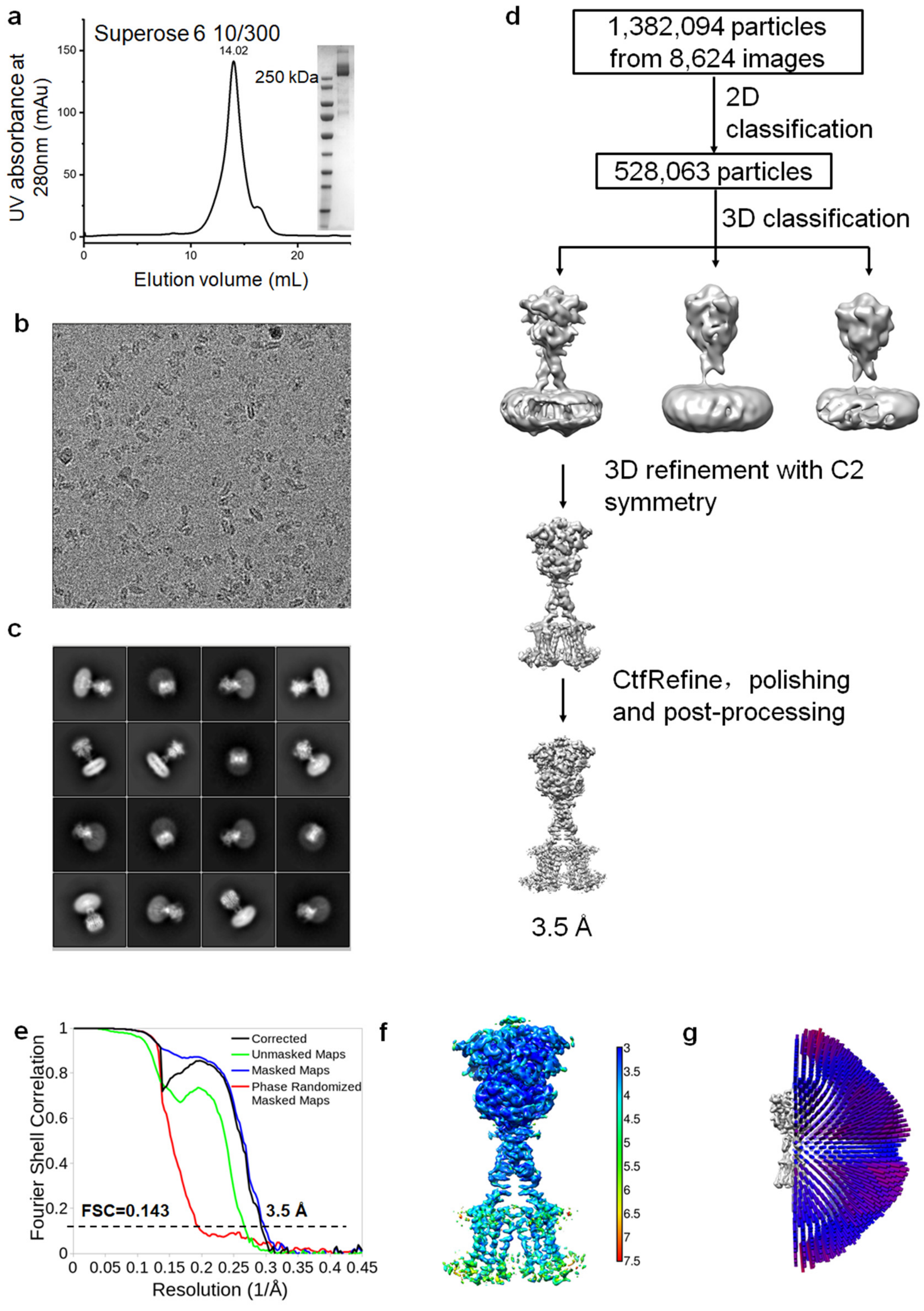

Fig. S1 Cryo-EM structure determination of CaSR in an active state. **a** Gel filtration and SDS-PAGE analysis of CaSR protein in DDM/CHS micelles. **b** A representative cryo-EM micrograph of CaSR in the Ca^{2+} and L-Trp-bound active state. **c** Representative 2D class averages of the active CaSR. **d** Cryo-EM data processing flow chart of CaSR in the Ca^{2+} and L-Trp-bound active state ($CaSR^{Acc}$). **e** Solvent-corrected Fourier shell correlation curve from Relion indicates that the resolution of the map is 3.5 Å at FSC = 0.143. **f** Density map of $CaSR^{Acc}$ colored according to local resolution estimation. **g** Particle angular distribution of final cryo-EM reconstruction of $CaSR^{Acc}$.
