# Supplementary material for: Structural mechanism of cooperative activation of the human calcium-sensing receptor by Ca2+ ions and L-tryptophan
Source: Cell Res. 2021 Feb 18;31(4):383–94. doi: 10.1038/s41422-021-00474-0 (PMC8115157; doi:10.1038/s41422-021-00474-0)
Supplement: Supplementary file 2 — Supplementary information, Figure S2 [file 41422_2021_474_MOESM2_ESM.pdf]

Supplementary information, Figure S2

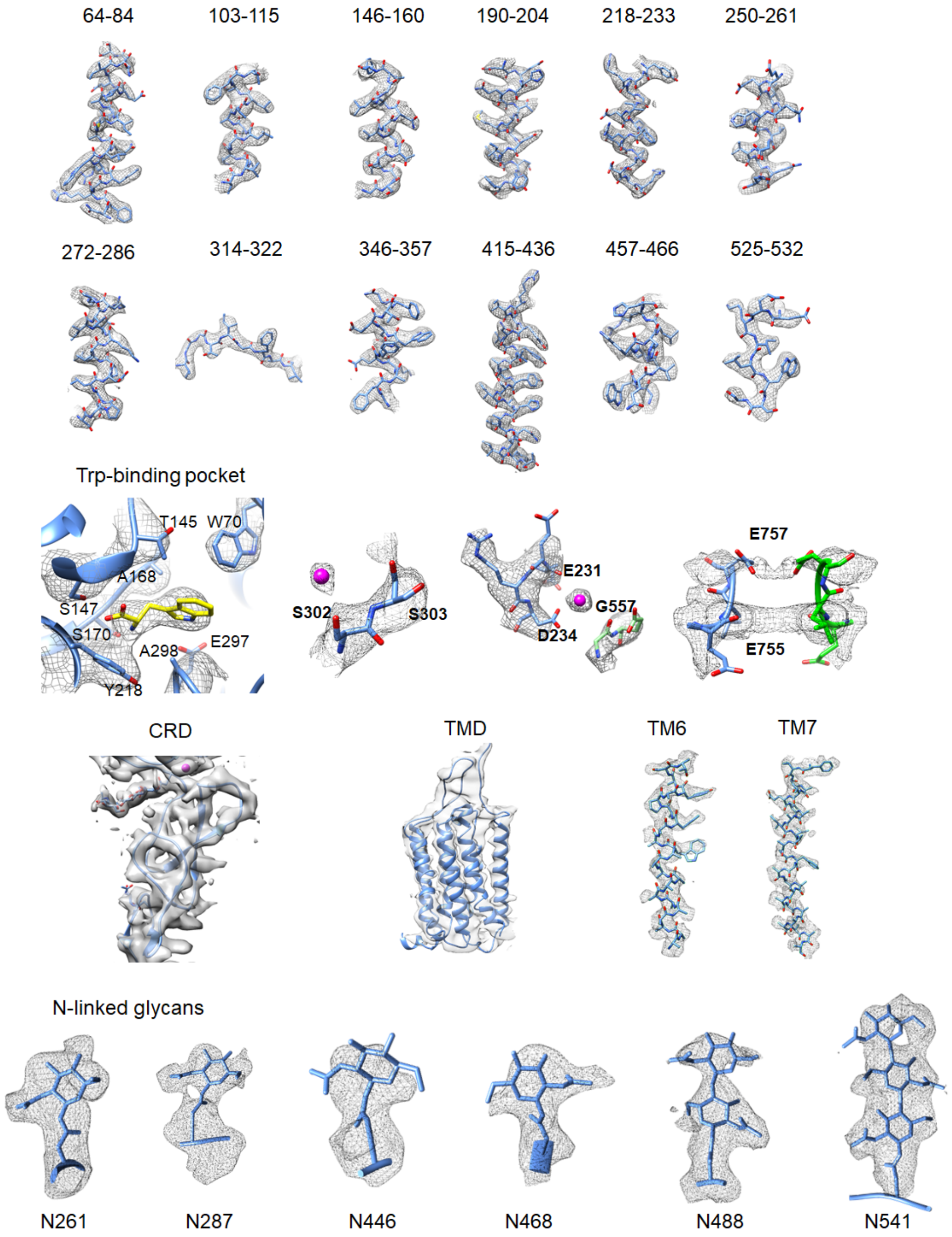

Fig. S2 Agreement between the cryo-EM map and the model of CaSR^{Acc}.

Representative cryo-EM densities and fitted atomic models for CaSR^{Acc}.
