# Supplementary material for: Structural mechanism of cooperative activation of the human calcium-sensing receptor by Ca2+ ions and L-tryptophan
Source: Cell Res. 2021 Feb 18;31(4):383–94. doi: 10.1038/s41422-021-00474-0 (PMC8115157; doi:10.1038/s41422-021-00474-0)
Supplement: Supplementary file 3 — Supplementary information, Figure S3 [file 41422_2021_474_MOESM3_ESM.pdf]

Supplementary information, Figure S3

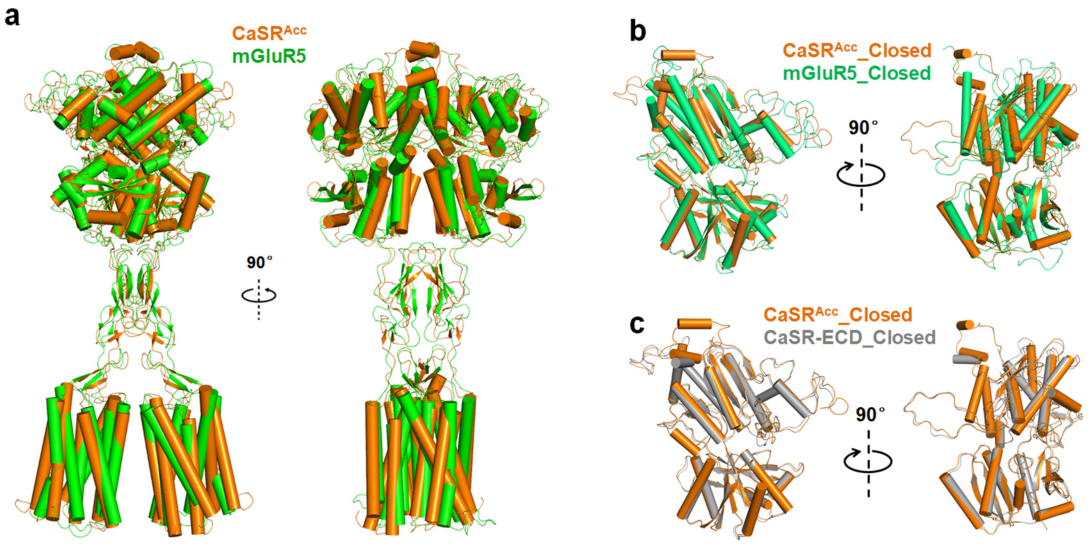

Fig. S3 Closed conformation of the VFT domain of CaSR^{Acc}. a Overall structural comparison between the full-length CaSR^{Acc} (orange) and mGluR5 (green, PDB code: 6N51) in active states. **b**, **c** Structure comparisons of VFT domains in closed conformation. The single VFT domain divided from the cryo-EM structure of full-length CaSR in an active closed-closed state (CaSR^{Acc}_Closed, orange) was aligned with that from the structure of full-length mGlu5 in an active state (PDB: 6N51) (**b**, mGluR5_Closed, green), and the structure of CaSR-ECD in an active state (PDB: 5K5S) (**c**, CaSR-ECD_Closed, grey), respectively.
