# Supplementary material for: Structural mechanism of cooperative activation of the human calcium-sensing receptor by Ca2+ ions and L-tryptophan
Source: Cell Res. 2021 Feb 18;31(4):383–94. doi: 10.1038/s41422-021-00474-0 (PMC8115157; doi:10.1038/s41422-021-00474-0)
Supplement: Supplementary file 4 — Supplementary information, Figure S4 [file 41422_2021_474_MOESM4_ESM.pdf]

Supplementary information, Figure S4

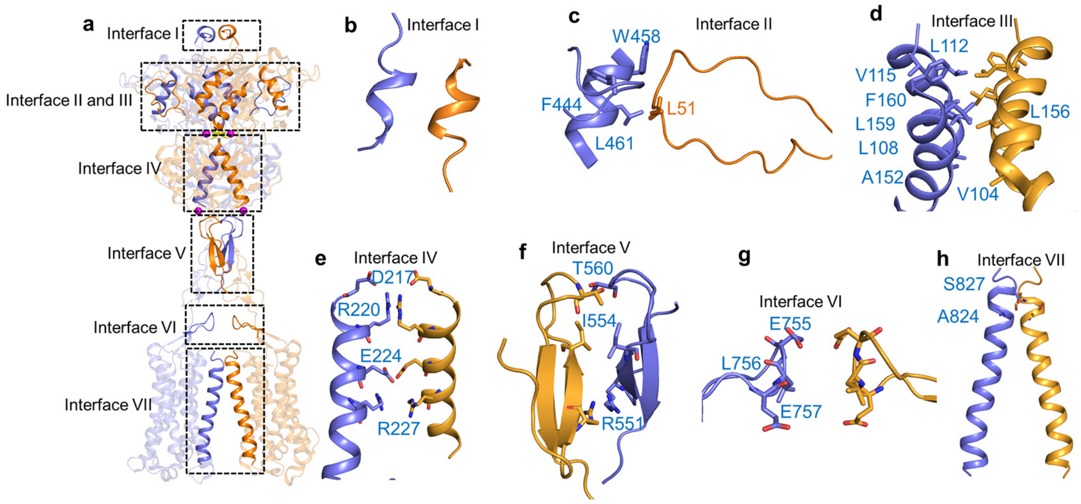

Fig. S4 Inter-subunit interfaces of the active CaSR homodimer. a Overall structure of the active CaSR showing seven inter-subunit interfaces, labeled as Interface I-VII. The two subunits of CaSR are colored in orange and slate. **b-h** Detailed views of the seven inter-subunit interfaces: “Interface I” around Cys129 in the apex region (**b**), “Interface II” between the LB1-loop and Helix-N in the adjacent subunit (**c**), “Interface III” between the Helix-B and Helix-D in the LB1 of the two subunit (**d**), “Interface IV” between Helix G in the LB2 of the two subunits (**e**), “Interface V” between the two CRDs (**f**), “Interface VI” between the second extracellular loops (ECL2) in the two TMDs (**g**), and “Interface VII” between the C terminal of TM6 of the active CaSR homodimer (**h**). Key residues mediating the contacts in each interface are indicated.
