# Supplementary material for: Structural mechanism of cooperative activation of the human calcium-sensing receptor by Ca2+ ions and L-tryptophan
Source: Cell Res. 2021 Feb 18;31(4):383–94. doi: 10.1038/s41422-021-00474-0 (PMC8115157; doi:10.1038/s41422-021-00474-0)
Supplement: Supplementary file 5 — Supplementary information, Figure S5 [file 41422_2021_474_MOESM5_ESM.pdf]

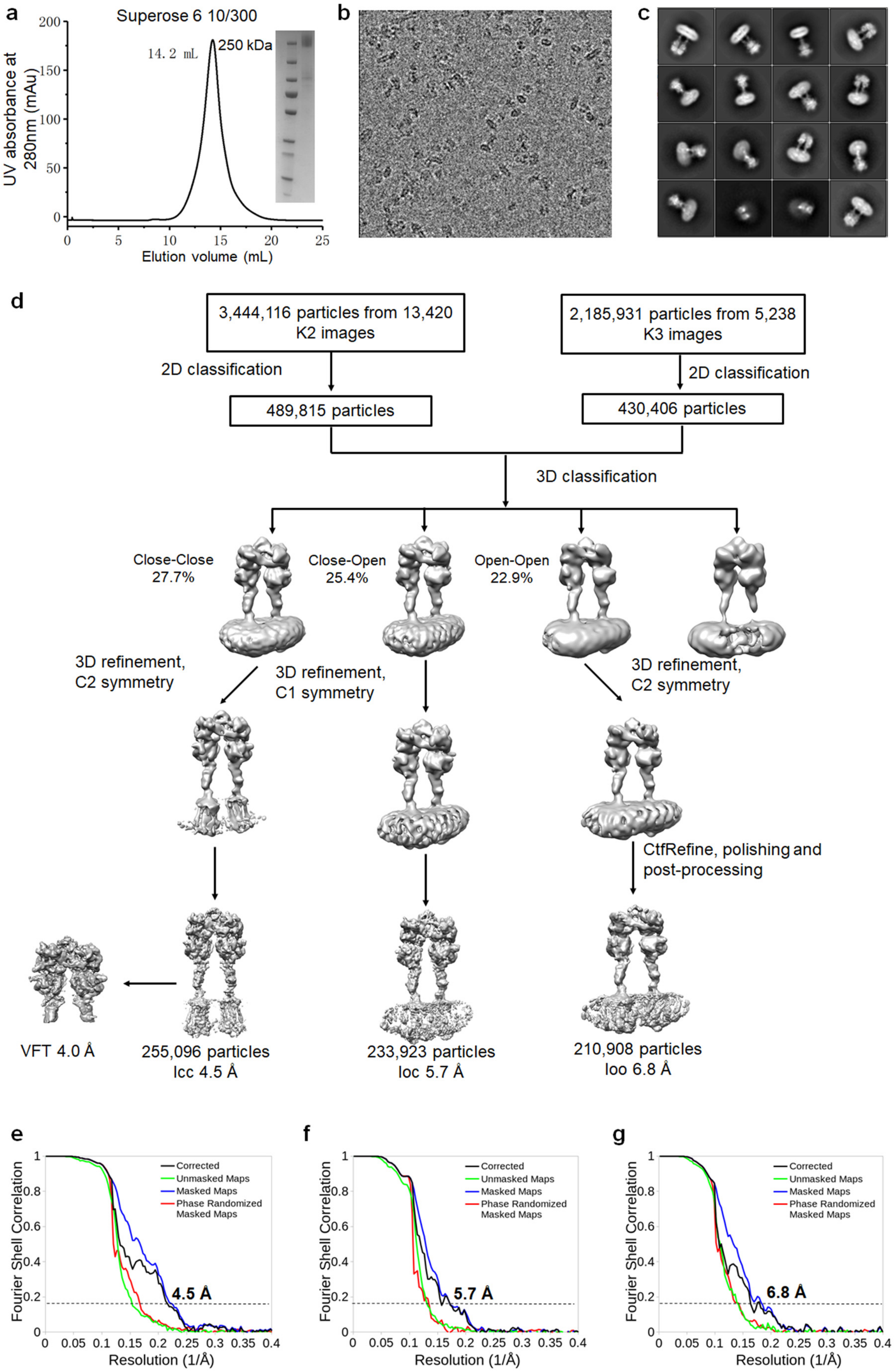

a
Superose 6 10/300
14.2 mL
250 kDa
UV absorbance at 280nm (mAu)
Elution volume (mL)
b
c
d
3,444,116 particles from 13,420 K2 images
2,185,931 particles from 5,238 K3 images
2D classification
2D classification
489,815 particles
430,406 particles
3D classification
Close-Close 27.7%
Close-Open 25.4%
Open-Open 22.9%
3D refinement, C2 symmetry
3D refinement, C1 symmetry
3D refinement, C2 symmetry
CtfRefine, polishing and post-processing
VFT 4.0 Å
255,096 particles
Icc 4.5 Å
233,923 particles
Ioc 5.7 Å
210,908 particles
Ioo 6.8 Å
e
f
g
Corrected
Unmasked Maps
Masked Maps
Phase Randomized Masked Maps
Fourier Shell Correlation
Resolution (1/Å)
4.5 Å
5.7 Å
6.8 Å

Fig. S5 Cryo-EM structure determination of inactive CaSR. a Gel filtration and SDS-PAGE analysis of CaSR protein in DDM/CHS micelles. **b** A representative cryo-EM micrograph of inactive CaSR. **c** Representative 2D class averages of the inactive CaSR. **d** Cryo-EM data processing flow chart of inactive CaSR. **e-g** Solvent-corrected Fourier shell correlation curve from Relion indicated that the resolution of CaSR maps were 4.5 Å for inactive closed-closed (Icc) conformation (**e**), 5.7 Å for inactive open-closed (Ioc) conformation (**f**) and 6.8 Å for inactive open-open (Ioo) conformation (**g**) at FSC = 0.143.
