# Supplementary material for: Structural mechanism of cooperative activation of the human calcium-sensing receptor by Ca2+ ions and L-tryptophan
Source: Cell Res. 2021 Feb 18;31(4):383–94. doi: 10.1038/s41422-021-00474-0 (PMC8115157; doi:10.1038/s41422-021-00474-0)
Supplement: Supplementary file 6 — Supplementary information, Figure S6 [file 41422_2021_474_MOESM6_ESM.pdf]

Supplementary information, Figure S6

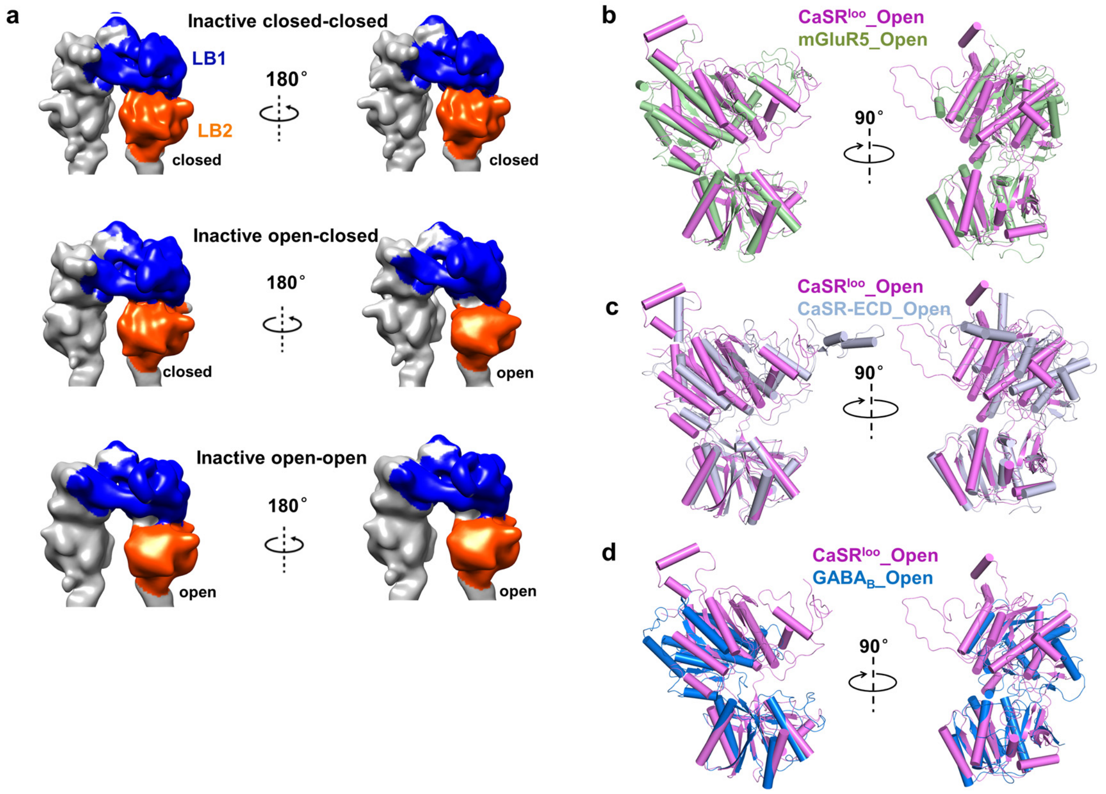

Fig. S6 Conformational heterogeneity of the VFT domains of CaSR in inactive state. a Three classes of 3D reconstruction density maps of the VFT domains of the inactive CaSR, showing conformational heterogeneity of the VFT domains (the LB1 and LB2 regions of each VFT domain are highlighted in blue and orange, respectively). The open or closed conformation of VFT is defined according to the widely separated LB1 and LB2 or tightly contacted LB1 and LB2. **b-d** Structure comparisons of VFT domains in open conformation. The single VFT domain divided from the cryo-EM structure of full-length CaSR in an inactive open-open state (CaSR^{Ioo}_Open, magenta) is aligned with that from the structure of full-length mGluR5 in an inactive state (PDB: 6N52) (**b**, mGluR5_Open, palegreen), the structure of CaSR-ECD in a resting state (PDB:5K5T) (**c**, CaSR-ECD_Open, bluewhite), and the structure of full-length GABA$_B$ in an inactive state (PDB: 7C7S) (**d**, GABA$_B$_Open, marine), respectively.
