# Supplementary material for: Structural mechanism of cooperative activation of the human calcium-sensing receptor by Ca2+ ions and L-tryptophan
Source: Cell Res. 2021 Feb 18;31(4):383–94. doi: 10.1038/s41422-021-00474-0 (PMC8115157; doi:10.1038/s41422-021-00474-0)
Supplement: Supplementary file 8 — Supplementary information, Figure S8 [file 41422_2021_474_MOESM8_ESM.pdf]

Supplementary information, Figure S8

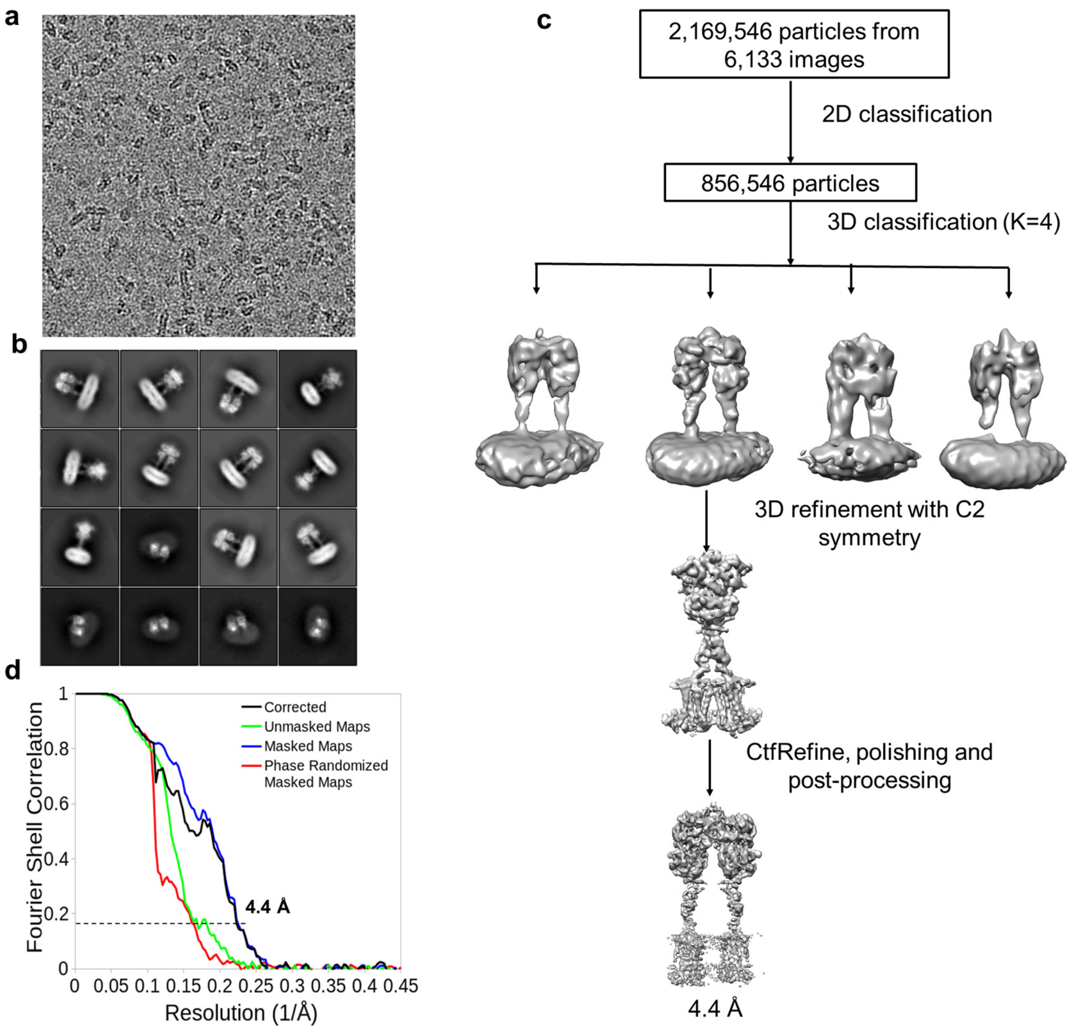

Fig. S8 Cryo-EM structure determination of CaSR in complex with L-Trp (CaSR^{Trp}). a A representative cryo-EM micrograph of CaSR^{Trp}. **b** Representative 2D class averages of the inactive CaSR^{Trp}. **c** Cryo-EM data processing flow chart of CaSR^{Trp}. **d** Solvent-corrected Fourier shell correlation curve from Relion indicated that the resolution of CaSR^{Trp} maps is 4.4 Å at FSC = 0.143.
