# Supplementary material for: Structural mechanism of cooperative activation of the human calcium-sensing receptor by Ca2+ ions and L-tryptophan
Source: Cell Res. 2021 Feb 18;31(4):383–94. doi: 10.1038/s41422-021-00474-0 (PMC8115157; doi:10.1038/s41422-021-00474-0)
Supplement: Supplementary file 9 — Supplementary information, Figure S9 [file 41422_2021_474_MOESM9_ESM.pdf]

Supplementary information, Figure S9

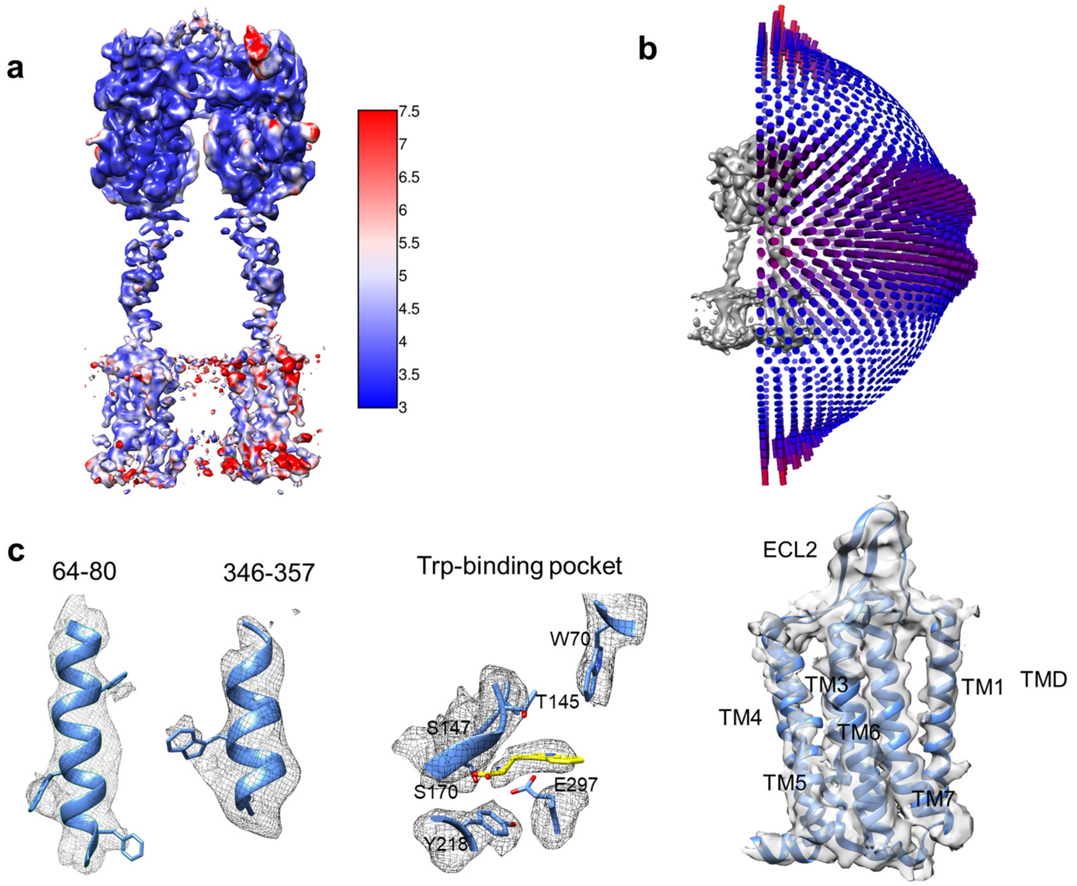

Fig. S9 Cryo-EM structure validation of CaSR^{Trp}. a Density map of CaSR^{Trp} was colored according to local resolution estimation. **b** Particle angular distribution of final cryo-EM reconstruction of CaSR^{Trp}. **c** Representative cryo-EM maps and fitted atomic models of CaSR^{Trp}, indicating the agreement between the cryo-EM map and the model.
