# Supplementary material for: Structural mechanism of cooperative activation of the human calcium-sensing receptor by Ca2+ ions and L-tryptophan
Source: Cell Res. 2021 Feb 18;31(4):383–94. doi: 10.1038/s41422-021-00474-0 (PMC8115157; doi:10.1038/s41422-021-00474-0)
Supplement: Supplementary file 10 — Supplementary information, Figure S10 [file 41422_2021_474_MOESM10_ESM.pdf]

Supplementary information, Figure S10

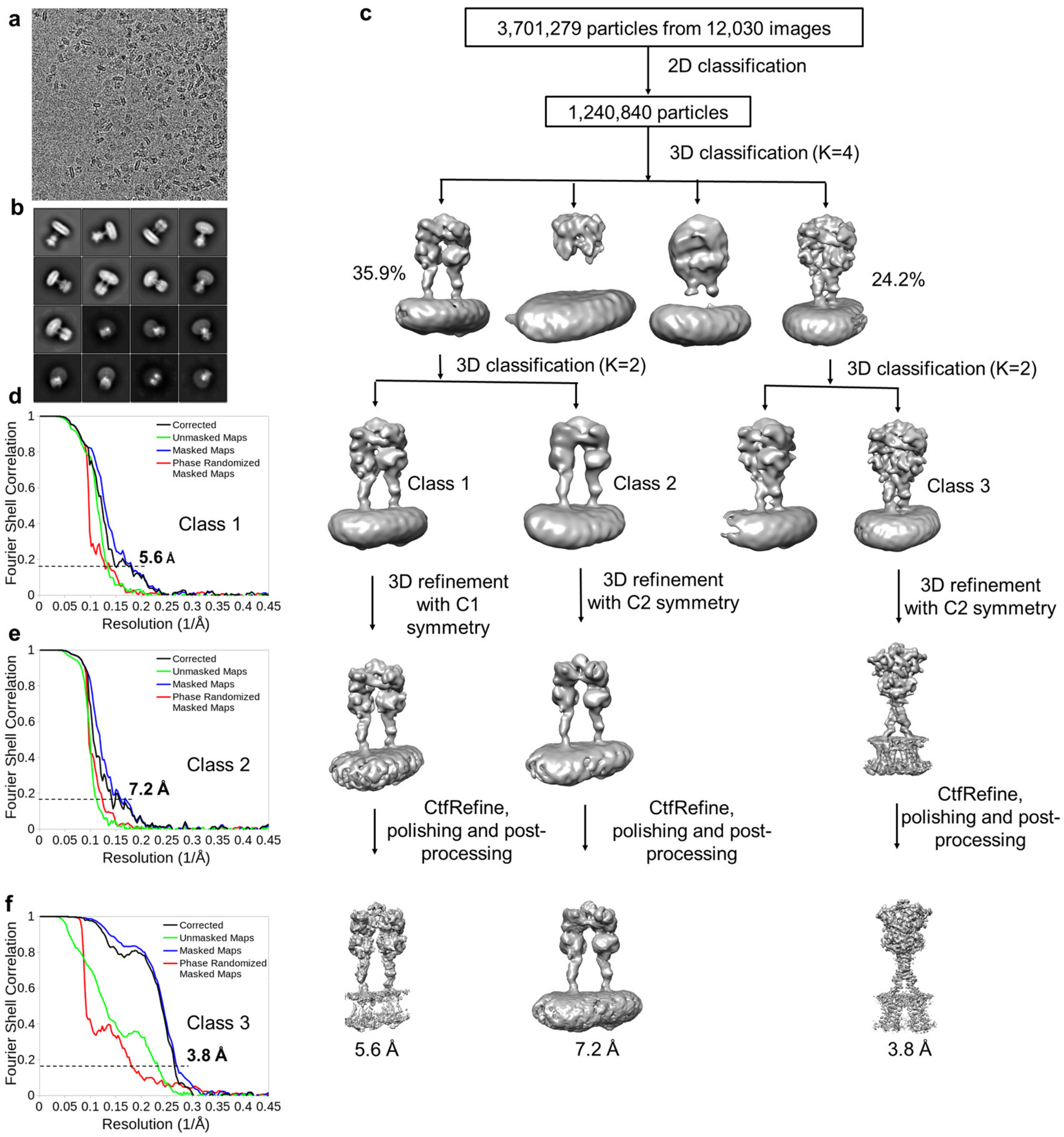

Fig. S10 Cryo-EM structure determination of CaSR supplemented with Ca^{2+} ions ($CaSR^{Ca}$). a A representative cryo-EM micrograph of $CaSR^{Ca}$. **b** Representative 2D class averages of $CaSR^{Ca}$. **c** Cryo-EM data processing flow chart of $CaSR^{Ca}$. **d-f** Solvent-corrected Fourier shell correlation curve from Relion indicated that the resolution of $CaSR^{Ca}$ maps were 5.6 Å, 7.2 and 3.8 Å for the three classes at FSC=0.143.
