# Supplementary material for: Structural mechanism of cooperative activation of the human calcium-sensing receptor by Ca2+ ions and L-tryptophan
Source: Cell Res. 2021 Feb 18;31(4):383–94. doi: 10.1038/s41422-021-00474-0 (PMC8115157; doi:10.1038/s41422-021-00474-0)
Supplement: Supplementary file 11 — Supplementary information, Figure S11 [file 41422_2021_474_MOESM11_ESM.pdf]

Supplementary information, Figure S11

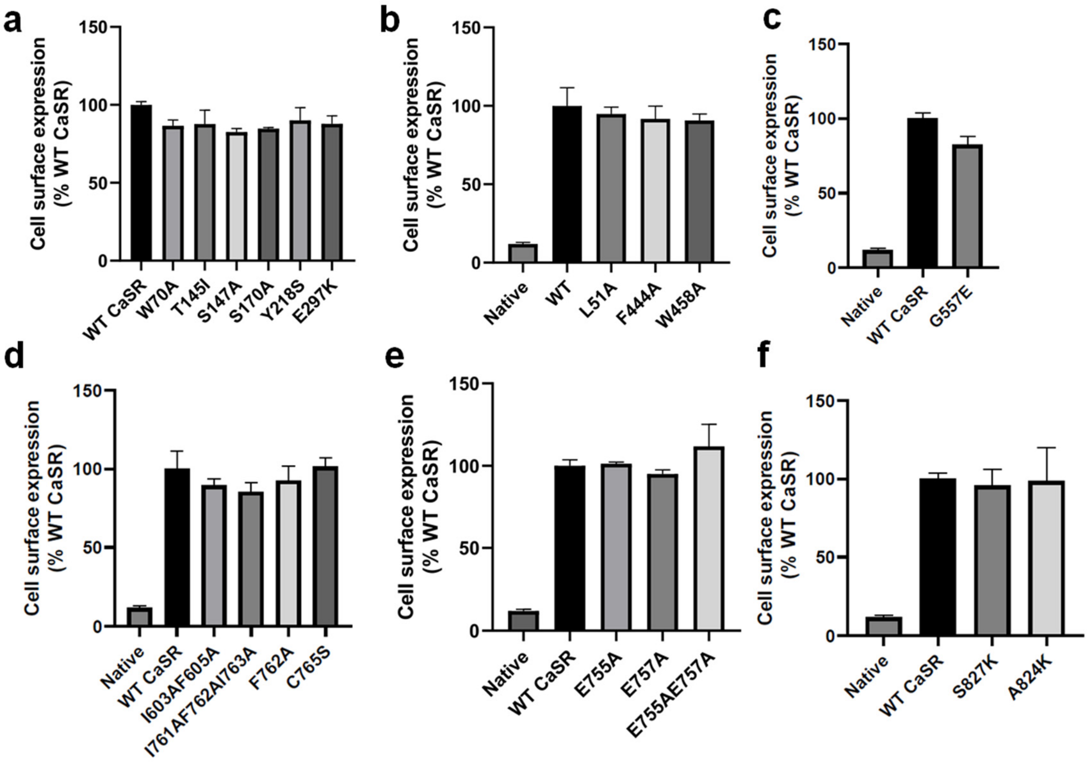

Fig. S11 Cell surface expression levels of wild-type CaSR and CaSR mutants. Cell surface expression of receptor mutants were determined by flow cytometry. Cell surface expression of each full-length CaSR mutant was not significantly different from the wild-type receptor. The data represent means ± SD from 3 independent experiments. Data were analyzed using one-way analysis of variance and Dunnett's post-test in which each mutant was compared to the wild-type receptor (WT CaSR).
