# Supplementary material for: Structural mechanism of cooperative activation of the human calcium-sensing receptor by Ca2+ ions and L-tryptophan
Source: Cell Res. 2021 Feb 18;31(4):383–94. doi: 10.1038/s41422-021-00474-0 (PMC8115157; doi:10.1038/s41422-021-00474-0)
Supplement: Supplementary file 12 — Supplementary information, Figure S12 [file 41422_2021_474_MOESM12_ESM.pdf]

Supplementary information, Figure S12

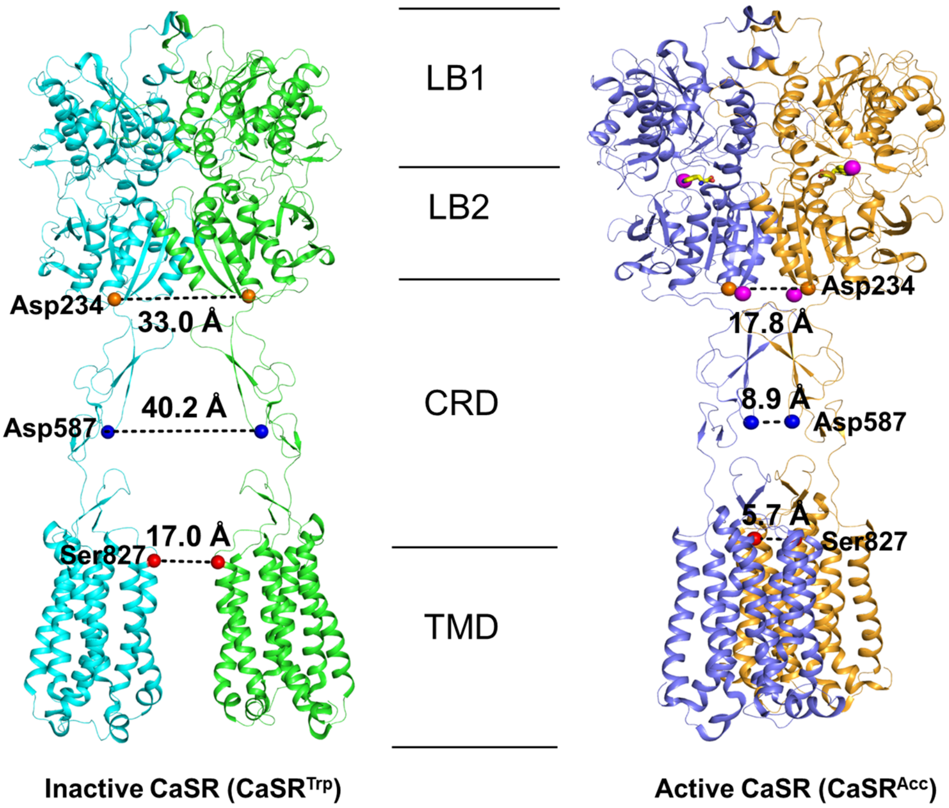

Fig. S12 Overall structural comparison of CaSR in inactive and active states. The structures of dimeric CaSR in L-Trp-bound inactive state ($CaSR^{Trp}$) and in Ca^{2+}/L-Trp-bound active ($CaSR^{Acc}$) are shown in cartoon representation. The two subunits of $CaSR^{Trp}$ are colored in green and cyan, and those of $CaSR^{Acc}$ are in orange and blue. Distances between Cα atoms of Asp234 in the two subunits (orange spheres), as well as Asp587 (blue) and Ser827 (red) are indicated.
