# Supplementary material for: Structural mechanism of cooperative activation of the human calcium-sensing receptor by Ca2+ ions and L-tryptophan
Source: Cell Res. 2021 Feb 18;31(4):383–94. doi: 10.1038/s41422-021-00474-0 (PMC8115157; doi:10.1038/s41422-021-00474-0)
Supplement: Supplementary file 13 — Supplementary information, Figure S13 [file 41422_2021_474_MOESM13_ESM.pdf]

Supplementary information, Figure S13

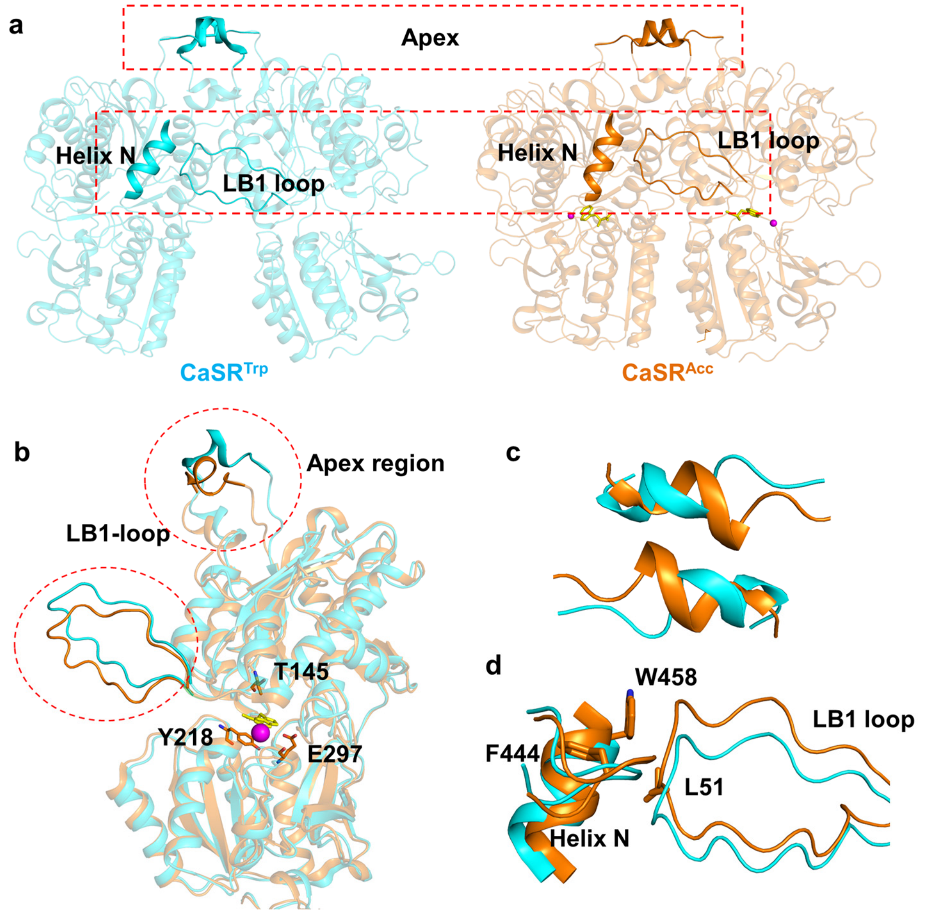

Fig. S13 Conformational changes of the interface between dimeric LB1 regions during CaSR activation. a Cartoon representation of the dimeric VFT domain structures of CaSR in an inactive (left, $CaSR^{Trp}$, cyan) and active state (right, $CaSR^{Acc}$, orange). The interfaces between the apex regions of the two subunits, and between the LB1 loop and Helix N in the adjacent subunits are indicated. **b** Structural superposition of a single VFT domain derived from the inactive closed-closed (cyan) and active (orange) CaSR structures. Significant conformational changes in the apex region and the LB1 loop are observed. Ca^{2+} is shown as a magenta sphere, and Trp as yellow stick. The residues coordinating the Trp are shown as orange sticks. **c, d** Detailed representation of the interfaces between the apex regions of the two subunits (**c**), and between the LB1 loop and the Helix N in the adjacent subunit. The dimeric VFT domains derived from the inactive (cyan) and active (orange) CaSR structures are aligned.
