# Supplementary material for: Structural mechanism of cooperative activation of the human calcium-sensing receptor by Ca2+ ions and L-tryptophan
Source: Cell Res. 2021 Feb 18;31(4):383–94. doi: 10.1038/s41422-021-00474-0 (PMC8115157; doi:10.1038/s41422-021-00474-0)
Supplement: Supplementary file 14 — Supplementary information, Figure S14 [file 41422_2021_474_MOESM14_ESM.pdf]

Supplementary information, Figure S14

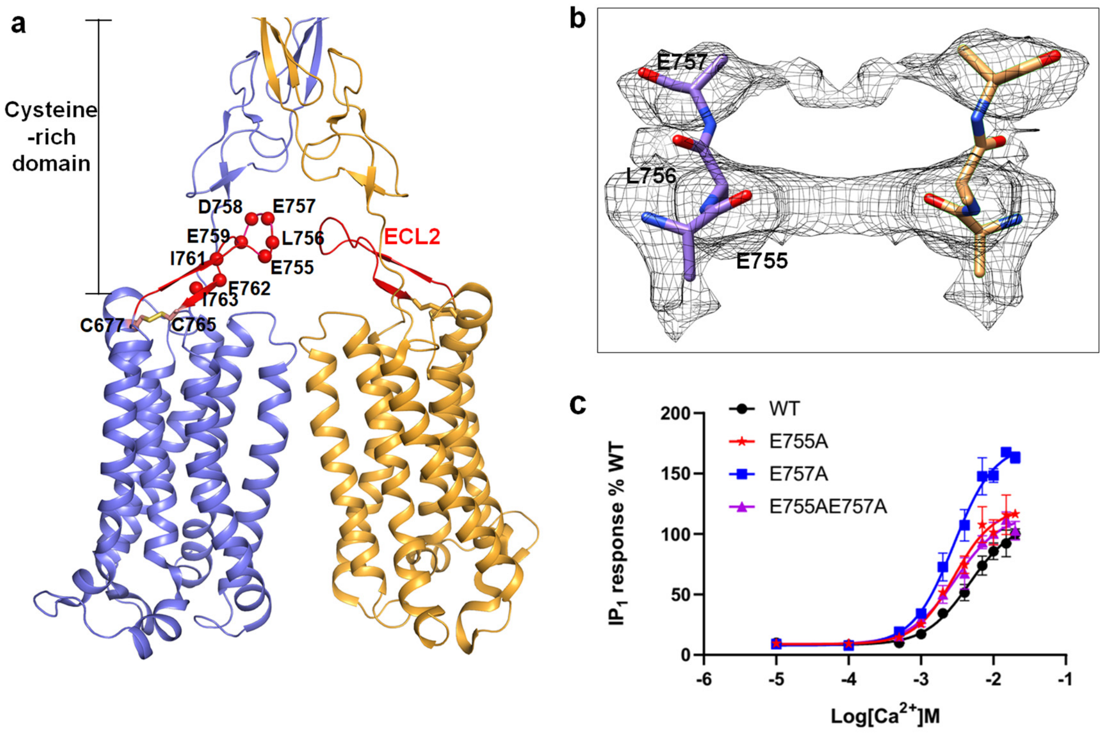

Fig. S14 Interaction between the ECL2 regions of the two subunits of $CaSR^{Acc}$. a Overall view of the TMD-ECL2 interactions. ECL2 and the linker region (569-610) connecting the CRD and TMD, and ECL2 of the two subunits are in close proximity. The Cα atoms of the residues in the ECL2 are shown as spheres. **b** The density map indicated a putative cation-binding site coordinated by two backbone carbonyl oxygen atoms of E755 at the interface formed between residues located at the tip region of ECL2 of each subunit. Residues E755, L756 and E757 are shown in sticks with their side chains omitted. **c** Site mutations of the putative Ca^{2+} binding site were observed to result in increased sensitivity of CaSR in the Ca^{2+}-stimulated IP1 accumulation assay. Single-site mutant E755A and E757A, double mutant E755A-E757A all showed increased sensitivity to Ca^{2+}. Data represent means ± SD of three independent experiments performed in triplicates.
