# Supplementary material for: Structural mechanism of cooperative activation of the human calcium-sensing receptor by Ca2+ ions and L-tryptophan
Source: Cell Res. 2021 Feb 18;31(4):383–94. doi: 10.1038/s41422-021-00474-0 (PMC8115157; doi:10.1038/s41422-021-00474-0)
Supplement: Supplementary file 15 — Supplementary information, Figure S15 [file 41422_2021_474_MOESM15_ESM.pdf]

Supplementary information, Figure S15

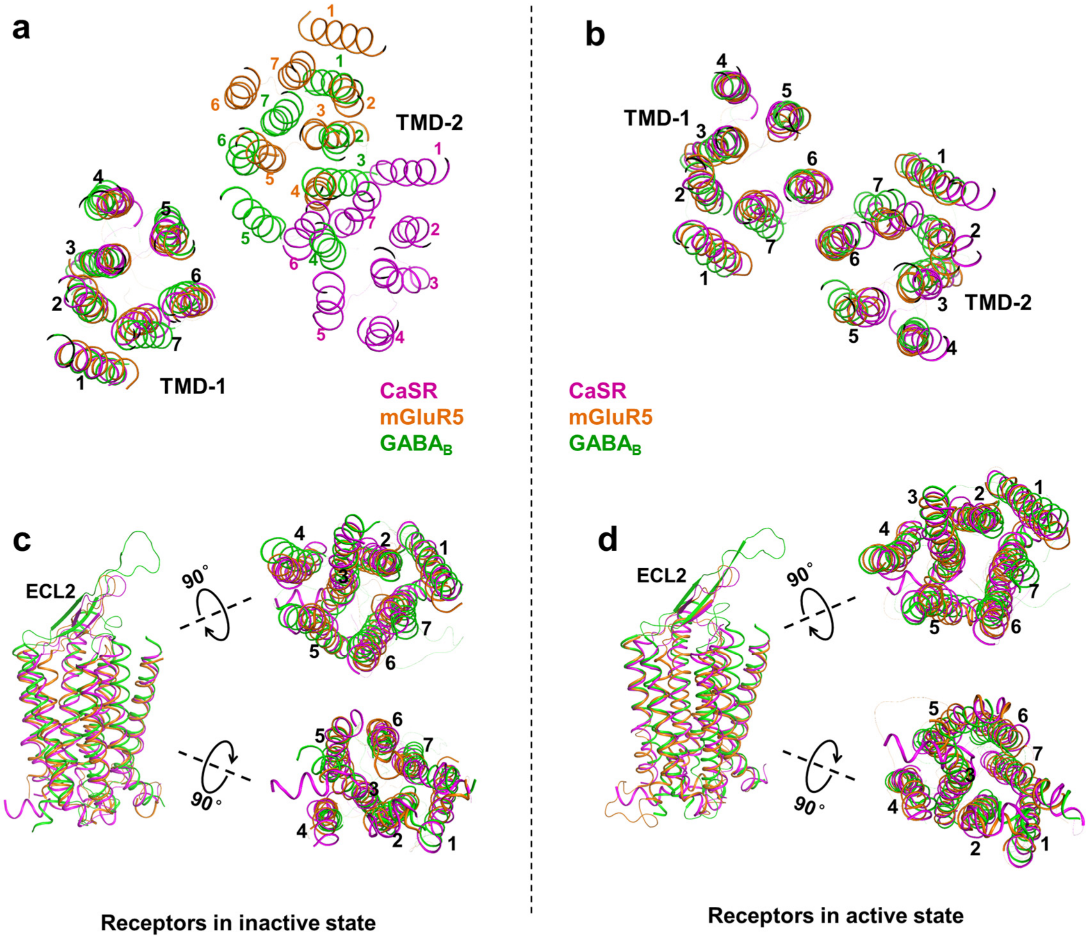

Fig. S15 Structural comparison of TMD of CaSR and TMDs of mGluR5 and $GABA_B$ receptors. a, b Extracellular view of the superposed dimeric TM bundles of CaSR (magenta), $GABA_B$ (green) and mGluR5 (orange) in inactive state (**a**) and active state (**b**). The TMD-1 of the three receptors are aligned. **c, d** Superimposition of a single TM domain divided from CaSR (magenta), GABAB (green) and mGluR5 (orange) in inactive state (**c**) and active state (**d**). PDB codes: inactive mGluR5, 6N52; active mGluR5, 6N51; inactive GABAB, 7C7S; active CaSR, 7C7Q.
