# Supplementary material for: Structural mechanism of cooperative activation of the human calcium-sensing receptor by Ca2+ ions and L-tryptophan
Source: Cell Res. 2021 Feb 18;31(4):383–94. doi: 10.1038/s41422-021-00474-0 (PMC8115157; doi:10.1038/s41422-021-00474-0)
Supplement: Supplementary file 16 — Supplementary information, Figure S16 [file 41422_2021_474_MOESM16_ESM.pdf]

Supplementary information, Figure S16

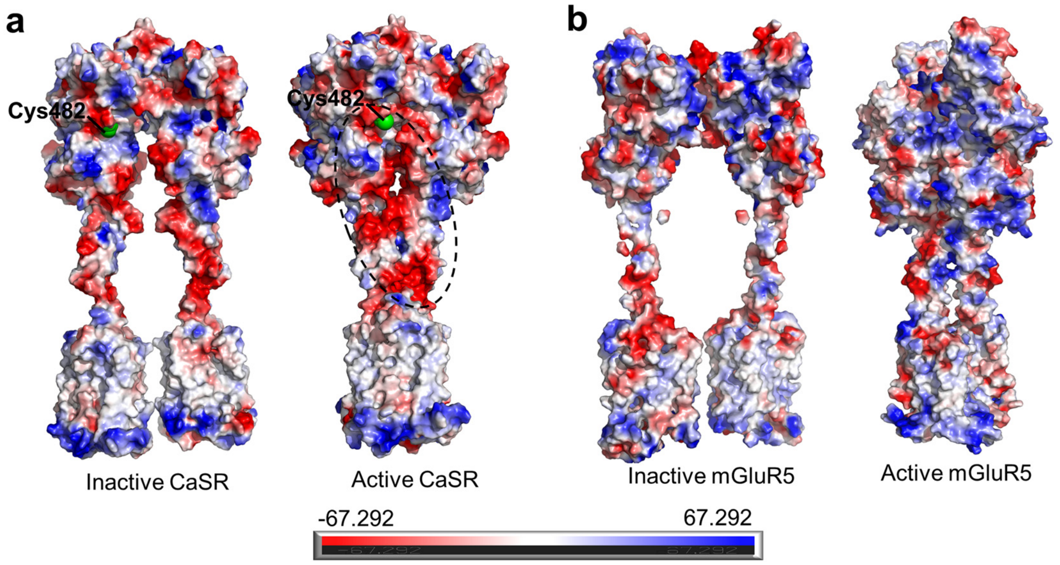

Fig. S16 Difference of electrostatic potential surfaces between CaSR and mGluR5.

a A negatively charged patch was observed connecting the LB2-dimer interface and CRD interface in the activate CaSR. Residue Cys482 is shown as green sphere. **b** Discrete distribution of charge residues at the dimer interface of CRD and LB2 domains in active mGluR5.
