# Supplementary material for: Structural mechanism of cooperative activation of the human calcium-sensing receptor by Ca2+ ions and L-tryptophan
Source: Cell Res. 2021 Feb 18;31(4):383–94. doi: 10.1038/s41422-021-00474-0 (PMC8115157; doi:10.1038/s41422-021-00474-0)
Supplement: Supplementary file 17 — Supplementary information, Table S1 [file 41422_2021_474_MOESM17_ESM.pdf]

Table S1 Cryo-EM data collection, structure refinement and validation statistics

	$CaSR^{Acc}$ (EMDB-30855) (PDB 7DTV)	$CaSR^{Ca}$ (EMDB-30853) (PDB 7DTT)	$CaSR^{Trp}$ (EMDB-30854) (PDB 7DTU)	$CaSR^{Icc}$ (EMDB-30856) (PDB 7DTW)	$CaSR^{Ioc}$ (EMDB-30858)	$CaSR^{Ioo}$ (EMDB-30857)
**Data collection and processing**						
Magnification	29,000	29,000	29,000	29,000/22,500*	29,000/22,500*	29,000/22,500*
Voltage (kV)	300	300	300	300	300	300
Electron exposure ($e^{-}/Å^2$)	62	62	56.5	62/60*	62/60*	62/60*
Defocus range (μm)	-1.3~-2.5	-1.3~-2.5	-1.3~-2.2	-1.3~-2.5	-1.3~-2.5	-1.3~-2.5
Pixel size (Å)	1.014	1.014	1.01	1.014/1.06*	1.014/1.06*	1.014/1.06*
Symmetry imposed	C2	C2	C2	C2	C1	C2
Initial particle images (no.)	1,382,094	3,701,279	2,169,546	3,444,116 /2,185,931*	3,444,116 /2,185,931*	3,444,116 /2,185,931*
Final particle images (no.)	229,926	315,050	240,292	255,096	233,923	210,908
Map resolution (Å)	3.5	3.8	4.4	4.5	5.7	6.8
FSC threshold	0.143	0.143	0.143	0.143	0.143	0.143
**Refinement**						
Initial model used (PDB code)	5k5s (ECD), 6n51(TMD)					
Model resolution (Å)	3.5	3.8	4.4	4.8		
	0.5	0.5	0.5	0.5		
FSC threshold						
Map sharpening *B* factor ($Å^2$)						
Model composition						
Non-hydrogen atoms	11,276	11,204	10,846	10,250		
Protein residues	1558	1558	1556	1556		
Ligands	2 Trp, 4 Ca^{2+}	4 Ca^{2+}	2 Trp			
B factors ($Å^2$)						
Protein	66.81	80.85	163.83	173.14		
Ligand	17.74 (Trp) 65.92 (Ca^{2+})	104.18 (Ca^{2+})	118.36 (Trp)			
R.m.s. deviations						
Bond lengths (Å)	0.008	0.011	0.010	0.007		
Bond angles (°)	1.005	1.149	0.933	1.056		
Validation						

MolProbity score	1.82	1.82	1.91	2.04
Clashscore	5.20	5.19	6.90	8.97
Poor rotamers (%)	0.00	0.00	0.00	0.00
Ramachandran plot				
Favored (%)	90.08	90.14	90.58	89.74
Allowed (%)	9.79	9.73	9.42	10.26
Disallowed (%)	0.13	0.13	0.00	0.00

*Two portions of inactive CaSR images were collected on a 300 kV Titan Krios equipped with a Gatan K2 Summit direct detection camera, and a 300 kV Titan Krios equipped with a Gatan K3 camera, with different magnifications and total electron dose. See Materials and Methods section for more information.
