# Supplementary material for: Structural mechanism of cooperative activation of the human calcium-sensing receptor by Ca2+ ions and L-tryptophan
Source: Cell Res. 2021 Feb 18;31(4):383–94. doi: 10.1038/s41422-021-00474-0 (PMC8115157; doi:10.1038/s41422-021-00474-0)
Supplement: Supplementary file 18 — Supplementary information, Table S2 [file 41422_2021_474_MOESM18_ESM.pdf]

Table S2. Summary of EC_{50} values, Emax and Hillslope for the wild-type CaSR and CaSR mutants. Curve-fitting was performed using a four-parameter logistic curve using the GraphPad prism. pEC_{50} values represent the negative logarithm of agonist concentration that produces half maximal response. Emax values are maximal response as percentage of wild-type CaSR response. All values are expressed as means ± SEM of three independent experiments conducted in triplicate. Data were performed using one-way analysis of variance followed by Dunnett's test using wild-type receptors as the control (**P* < 0.05, ***P* < 0.01, ****P* < 0.001, *****P* < 0.0001). n.d., not determined. n.s. no significantly different.

CaSR	EC_{50} (mM)	pEC_{50}	Dunnett's test	Emax	Dunnett's test	Hill-Slope	Dunnett's test
Wide Type	4.43±0.10	2.35±0.01		100±2.94		1.38±0.11	
W70A	n.d.	n.d.	n.d.	28.10±1.04	****	1.64±0.04	**
T145I	17±4.2	1.79±0.12	****	25.82±1.79	****	1.40±0.33	ns
S147A	n.d.	n.d.	n.d.	35.12±1.12	****	1.36±0.13	ns
Y218S	n.d.	n.d.	n.d.	32.49±1.30	****	1.22±0.05	*
S170A	16.49±2.7	1.79±0.07	****	45.27±0.51	****	2.30±0.29	***
E297K	n.d.	n.d.	n.d.	30.53±2.77	****	1.28±0.04	ns
L51A	5.95±0.06	2.23±0.004	****	66.01±2.42	***	1.57±0.08	*
F444A	5.16±0.16	2.29±0.014	**	110.8±4.63	ns	1.74±0.14	***
W458A	11.66±0.27	1.93±0.01	****	87.52±3.20	*	1.09±0.21	****
G557E	5.66±0.23	2.25±0.05	**	105.5±1.65	ns	2.88±0.20	****
F762A	6.29±0.41	2.20±0.03	****	87.61±3.06	*	1.34±0.05	ns
C765S	n.d.	n.d.	n.d.	43.05±1.49	****	2.16±0.71	*
I603AF605A	5.35±0.61	2.28±0.05	*	110.0±1.80	*	1.48±0.12	ns
I761AF762AI763A	16.32±1.79	1.79±0.05	****	47.20±1.27	****	1.34±0.06	ns
A824K	12.06±0.88	1.92±0.03	****	28.97±1.17	****	1.00±0.03	****
S827K	9.36±0.16	2.03±0.007	****	89.55±2.58	ns	2.64±0.01	****
